# Functional integration of the circulatory, immune, and respiratory systems in mosquito larvae: pathogen killing in the hemocyte-rich tracheal tufts

**DOI:** 10.1186/s12915-016-0305-y

**Published:** 2016-09-19

**Authors:** Garrett P. League, Julián F. Hillyer

**Affiliations:** Department of Biological Sciences, Vanderbilt University, VU Station B 35-1634, Nashville, TN 37235 USA

**Keywords:** Phagocytosis, Melanization, Trachea, Heart, Hemolymph, Circulation, *Anopheles gambiae*, Larva, Insect

## Abstract

**Background:**

﻿As both larvae and adults, mosquitoes encounter a barrage of immune insults, ranging from microbe-rich communities in larval habitats to ingested blood-borne pathogens in adult blood meals. Given that mosquito adults have evolved an efficient means of eliminating infections in their hemocoel (body cavity) via the coordinated action of their immune and circulatory systems, the goal of the present study was to determine whether such functional integration is also present in larvae.

**Results:**

By fluorescently labeling hemocytes (immune cells), pericardial cells, and the heart, we discovered that fourth instar larvae, unlike adults, contain segmental hemocytes but lack the periostial hemocytes that surround the ostia (heart valves) in abdominal segments 2–7. Instead, larvae contain an abundance of sessile hemocytes at the tracheal tufts, which are respiratory structures that are unique to larvae, are located in the posterior-most abdominal segment, and surround what in larvae are the sole incurrent openings for hemolymph entry into the heart. Injection of fluorescent immune elicitors and bacteria into the larval hemocoel then showed that tracheal tuft hemocytes mount rapid and robust immune responses against foreign insults. Indeed, green fluorescent protein-labeled *Escherichia coli* flowing with the hemolymph rapidly aggregate exclusively at the tracheal tufts, where they are killed within 24 h post-infection via both phagocytosis and melanization.

**Conclusion:**

Together, these findings show that the functional integration of the circulatory, respiratory, and immune systems of mosquitoes varies drastically across life stages.

**Electronic supplementary material:**

The online version of this article (doi:10.1186/s12915-016-0305-y) contains supplementary material, which is available to authorized users.

## Background

Insects have evolved powerful innate immune responses to neutralize infectious agents [1, 2]. Pathogens, including bacteria, viruses, malaria parasites, and fungi, invade the insect hemocoel (body cavity) after ingestion and penetration of the midgut, through breaches in the cuticle, or through the tracheal system. Upon entering the hemocoel, cellular and humoral immune factors kill pathogens by phagocytosis, melanization, lysis, and other mechanisms [2, 3]. The primary immune cells that drive these processes are the hemocytes, which are found both circulating with the hemolymph and attached to tissues (sessile) [4–6].

Immune responses in the hemocoel occur in a dynamic space, where hemocytes, humoral immune factors, and pathogens are propelled through the body by the action of an open circulatory system [2, 7]. This circulation is primarily mediated by the contractile action of a muscular dorsal vessel that comprises two distinct regions: the abdominal heart and the thoracic aorta [1, 8]. Because circulatory currents move both pathogens and immune components, functional integration is expected to exist between the immune and circulatory systems. Such integration was recently documented in the adult stage of the malaria mosquito *Anopheles gambiae*, where cellular immune responses are rapid and inducible on the surface of the heart [9]. Specifically, in adult mosquitoes, hemolymph enters the lumen of the dorsal vessel via valves, called ostia, that are located in the anterior portion of abdominal segments 2–7 [10, 11]. A population of sessile hemocytes, called periostial hemocytes, is always present on the surface of the heart in the regions that flank the ostia, where they phagocytose pathogens as they are swept with the hemolymph toward the heart [9, 12, 13]. This initial capture of circulating pathogens induces the migration of additional hemocytes to each periostial region, where they aggregate with the initial population of first responders and amplify the phagocytosis response. Although heart-associated immune aggregations have been observed in multiple mosquito studies [9, 12–19], they are not unique to this group of insects. Such aggregations have also been directly or indirectly observed in *Drosophila melanogaster* adults [20–27], though these studies did not seek to link immune responses with the functional mechanics of the heart or with hemolymph flow.

**Fig. 1:**
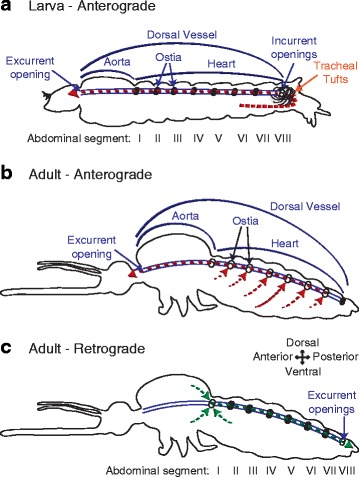
Hemolymph flow through the larval and adult dorsal vessels changes during development. **a** Lateral view of a larva showing the dorsal vessel with ostia. Hemolymph enters the dorsal vessel via a pair of incurrent openings at the posterior heart terminus and exits into the head via an excurrent opening. The larval heart only contracts anterograde. **b** Lateral view of an adult mosquito during an anterograde heart contraction period. Hemolymph enters the dorsal vessel via paired ostia in abdominal segments 2–7 and exits into the head via an excurrent opening. **c** Lateral view of an adult mosquito during a retrograde heart contraction period. Hemolymph enters the dorsal vessel via a pair of ostia at the thoraco-abdominal junction and exits the heart via a pair of excurrent openings at the posterior heart terminus. Illustrations are not drawn to scale. The movement of hemolymph is illustrated using *dashed arrows*

To date, no study has examined whether similar interactions between the immune and circulatory systems occur in insect larvae. However, prior to the initiation of this work, several lines of evidence suggested that any such interactions, if they were to exist in mosquito larvae, would differ from what is seen in adults [10, 11]. First, intracardiac hemolymph in larvae flows solely in the anterograde direction (toward the head), as the larval heart, unlike the adult heart, does not undergo heartbeat directional reversals (Fig. 1). Second, although the ostia of larvae and adults are similar in number, structure, and position, larval ostia are inert and do not serve as incurrent (inflow) valves for hemolymph entry into the heart (Fig. 1a, b). Third, despite sharing a similar structure, the posterior terminus of the larval heart serves as the sole incurrent opening for hemolymph entry into the dorsal vessel, whereas this same posterior terminus in adults only has excurrent (outflow) function (Fig. 1a, c).

In the present study we assessed whether the circulatory and immune systems of mosquito larvae interact during the course of an infection, and describe a pattern that is remarkably different from what is seen in adults. Specifically, we show that, unlike in adults, infection of a mosquito larva does not induce the aggregation of hemocytes and pathogens in the periostial regions of the heart. Instead, infection induces the rapid aggregation and killing of pathogens on the surface of the tracheal tufts, which are respiratory structures that are unique to larvae and associate with the posterior terminus of the heart in the eighth abdominal segment. In a process that is functionally analogous to what is seen in the periostial regions of adults, the tracheal tuft hemocytes of larvae phagocytose pathogens as they near entry into the dorsal vessel, and thus sequester and kill pathogens in the area of the body with the highest hemolymph flow. Taken together, these findings show that the functional integration of the circulatory and immune systems of mosquitoes displays drastic stage-specific differences that reflect the broad changes in body plan that occur during metamorphosis.

## Results

### Larval sessile hemocytes are segmentally arranged and enriched in the eighth abdominal segment

**Fig. 2:**
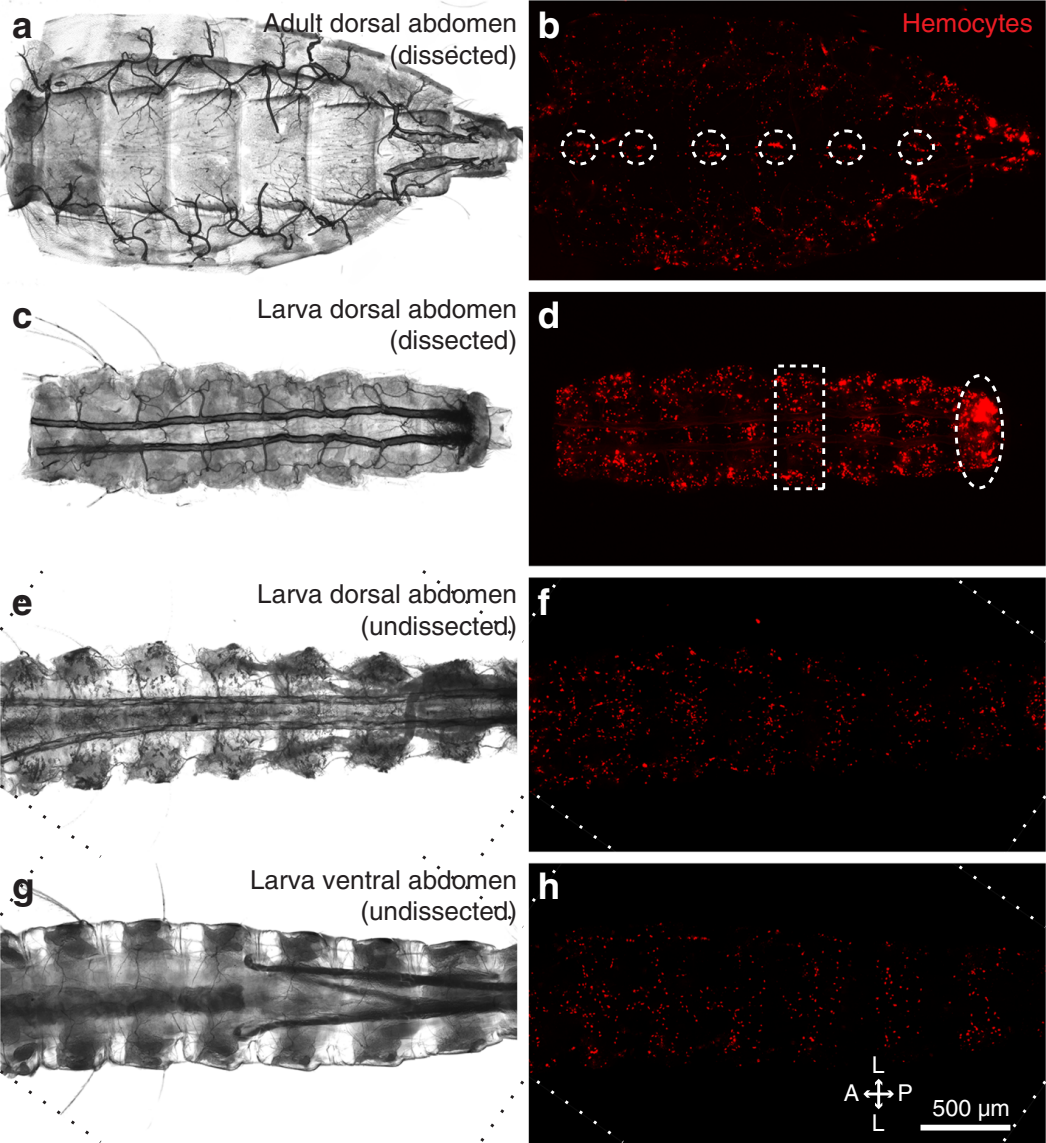
Sessile hemocyte distributions in the abdomen differ between larvae and adults. **a, b** Dissected adult dorsal abdomen under bright-field (**a**) and fluorescence illumination (**b**). Hemocytes (CM-DiI; *red*) form paired aggregates at the periostial regions of the heart (circles). **c, d** Dissected fourth instar larval dorsal abdomen under bright-field (**c**) and fluorescence illumination (**d**). Hemocytes form segmental bands in each abdominal segment (e.g., *rectangle*) and are also concentrated in the eighth abdominal segment (*oval*). **e–h** Undissected larval abdomen imaged from the dorsal (**e**, **f**) and ventral (**g**, **h**) views using bright-field (**e**, **g**) and fluorescence illumination (**f**, **h**). Hemocytes show a segmental banding pattern that encircles the abdomen in each abdominal segment. *Diagonal lines* in panels **e**–**h** denote the edges of rotated images. *Directional arrows*: *A* anterior, *P* posterior, *L* lateral

In adult mosquitoes, sessile hemocytes that are associated with the periostial regions of the heart work in concert with hemolymph flow to clear infections [9, 13]. To determine whether a similar phenomenon occurs in larvae, we initially injected fourth instar larvae and adult mosquitoes with CM-DiI cell labeling solution, which stains hemocytes [9, 12], and examined the distribution of these cells in the dorsal abdomen by fluorescence microscopy. Analyses of whole and dissected mosquitoes revealed that larvae and adults have a markedly different pattern of sessile hemocyte distribution (Fig. 2). Five-day-old adult females showed a dispersed distribution of hemocytes with no clear segmentation pattern other than the aggregation of hemocytes at the periostial regions of the heart (Fig. 2a, b). In contrast, larvae displayed dense bands of segmentally arranged hemocytes (Fig. 2c, d). These segmental hemocyte bands form continuous rings that encircle each abdominal segment, as this banding is identical in the dorsal and ventral halves of the body (Fig. 2c–h). Furthermore, larvae do not contain the distinct periostial hemocyte aggregates that are typically observed along the heart of adults. Instead, they contain a high concentration of hemocytes in the eighth abdominal segment, a region of the body that contains far fewer hemocytes in adults (Fig. 2b, d).

### The larval heart lacks periostial hemocytes

**Fig. 3:**
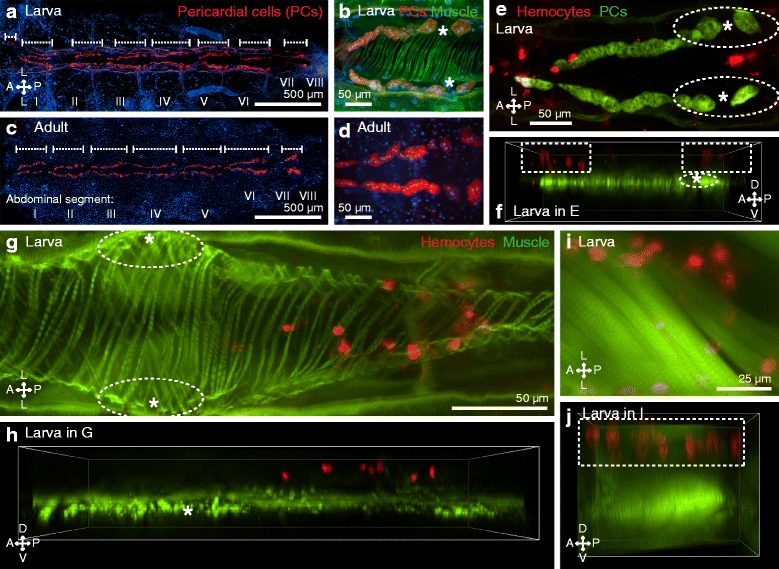
Larvae contain pericardial cells but lack periostial hemocytes. **a** Dissected larval dorsal abdomen with fluorescently labeled pericardial cells (IgG 568 nm; *red*). Nuclei were stained *blue* with Hoechst 33342, and the pericardial cell clusters are delineated by the *whiskers*. **b** A single set of pericardial cell clusters (*red*) of a larva, showing the large posterior-most pairs of cells and the gap that forms at a location that is just ventral of each ostium (*asterisk*) of the heart (phalloidin; *green*). **c, d** A dissected adult abdomen showing pericardial cell clusters in the same locations as in larvae. Images in panels **a** and **c** were assembled by stitching three higher resolution images. **e, f** Co-labeling of pericardial cells (IgG 488 nm; *green*) and hemocytes (CM-DiI; *red*) of a larva, displayed both as an Extended Depth of Focus (*EDF*) image (**e**) and a three-dimensional (*3D*) volume view image (**f**). Segmental hemocyte bands (*rectangles*) lie dorsal to the pericardial cells, and no hemocytes are present at the periostial regions (*oval*) that flank the ostia (**asterisks**). **g, h** EDF (**g**) and 3D volume view (**h**) fluorescence images showing that the segmental hemocytes (*red*) of a larva are dorsal to the heart (*green*) and distant from the ostia (*asterisks*). No hemocytes are present at the periostial regions (*ovals*). **i, j** EDF (**i**) and 3D volume view (**j**) fluorescence images of a larva showing that the segmental hemocytes (*red*; *rectangle*) lie dorsal to the swim muscles (*green*). *Directional arrows*: *A* anterior, *P* posterior, *D* dorsal, *V* ventral, *L* lateral

To confirm the absence of periostial hemocytes along the larval heart, hemocytes and either the heart or its closely associated pericardial cells, which we used to establish the location of the ostia, were examined by fluorescence microscopy. Injection of larvae with Alexa Fluor conjugated immunoglobulin G (IgG), which labels pericardial cells via their pinocytic function, revealed that the pericardial cells in this life stage flank the heart in a manner that is similar to what is seen in adults (Fig. 3a–d). These cells form seven paired clusters on either side of the ventrolateral surface of the heart, and each paired cluster spans an abdominal suture. In addition, larvae contain an additional paired cluster that has not been documented in adults, which consists of two pericardial cells on either side of the dorsal vessel and spans the thoraco-abdominal junction. With the exception of the pericardial cell clusters located in the eighth abdominal segment, the two posterior-most pairs of pericardial cells within each segmental cluster are larger and are spaced such that a small gap forms between each lateral pair along the anterior-posterior axis (Fig. 3b, d, e) [9, 11]. In both life stages, the ostia lie just dorsal to this small gap between the pericardial cells, and in adults, this gap is flanked by periostial hemocytes [9, 11, 13].

When the pericardial cells and hemocytes were co-stained, the periostial regions of the larval heart were devoid of hemocytes (Fig. 3e). Though at first glance some of the nearby segmentally arranged hemocytes appeared to occupy the periostial space, 3D deconvolved Z-stacks clearly showed that these segmental hemocytes are attached to the integument that is immediately underneath the cuticle, and thus, these cells lie far dorsal to the ostia, where the periostial hemocytes would be located (Fig. 3f). Staining of heart muscle with Alexa Fluor-conjugated phalloidin, which binds F-actin, in conjunction with hemocyte staining, confirmed this observation by revealing a distinct lack of periostial hemocytes in larvae (Fig. 3g, h). Instead, segmental hemocytes are present on the dorsal integument in a location that is dorsal to both the heart (Fig. 3g, h) and the surrounding swim muscles (Fig. 3i, j).

### Larval hemocytes densely populate the eighth abdominal segment tracheal tufts

**Fig. 4:**
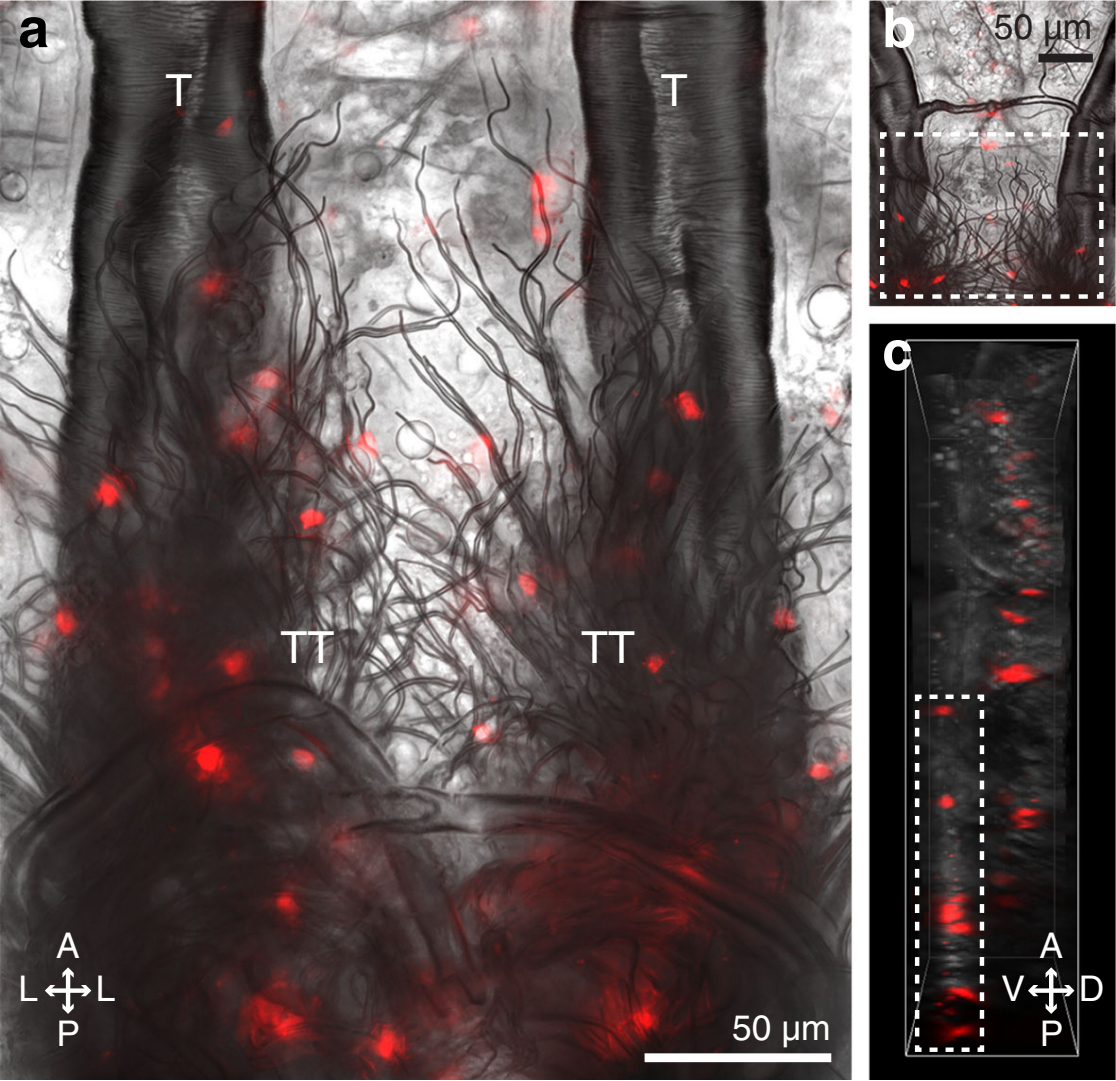
Larval hemocytes associate with the eighth abdominal segment tracheal tufts and are distinct from the segmental hemocyte bands. **a** Posterior portion of the dorsal abdomen of a dissected larva showing the trachea of the seventh and eighth abdominal segments under bright-field and fluorescence illumination. A dense mass of tracheoles known collectively as the tracheal tufts (*TT*) extend ventrally from the dorsal longitudinal tracheal trunks (*T*). Hemocytes (CM-DiI; *red*) bind to the tracheal tufts in high numbers. **b, c** EDF (**b**) and 3D volume view (**c**) images of a portion of the seventh and eighth abdominal segments of a larva. Tracheal tuft hemocytes (*rectangles*) lie ventral to the dorsal segmental hemocytes. *Directional arrows*: *A* anterior, *P* posterior, *D* dorsal, *V* ventral, *L* lateral


Qualitative observation of the entire length of mosquito larvae revealed that hemocytes are more abundant in the eighth abdominal segment than in any other region of the body, including the periostial regions (Fig. 2d). In larvae, the eighth abdominal segment is functionally analogous to the periostial regions, as it is the region of the body with the highest hemolymph flow and the only location where hemolymph enters the dorsal vessel [11, 13]. However, because the heart is narrow and terminates just posterior to the suture that joins the seventh and eighth abdominal segments [11], the diffuse spatial arrangement of these hemocytes led us to hypothesize that they are largely associated with non-cardiac tissue. A closer examination of the eighth abdominal segment of larvae revealed a dense population of sessile hemocytes that is bound to specialized tracheoles that are arranged into structures collectively referred to as the tracheal tufts (Fig. 4). Recently described in *A. gambiae* larvae [11], the eighth abdominal segment tracheal tufts are a dense network of thin tracheoles that emanate from the ventral base of the dorsal longitudinal tracheal trunks (Fig. 4a, b; Additional file 1: Figure S1A). Although these tracheoles are predominantly suspended in the hemolymph, some are attached to the posterior terminus of the heart, thus causing them to move with each heartbeat (Additional file 2: Movie S1). Furthermore, examination of the exuviae of fourth instar larvae and the dissected dorsal abdomens of pupae and adults showed that the tracheal tufts are unique to larvae and are shed during pupation (Additional file 1: Figure S1).

**Additional file 2 Movie S1.** The tracheal tufts move as a consequence of the wave-like contractions of the heart. Intravital video through the dorsal cuticle of the eighth abdominal segment of a larva showing that the tracheal tuft tracheoles move in concert with each contraction of the heart. Frame rate: real time.

The tracheal tuft hemocytes comprise a population of cells that is distinct from the segmental hemocytes, as the former are located ventral to the tracheal trunks, whereas the latter are attached to the integument (Fig. 4b, c). In addition to binding the tracheal tuft tracheoles, larval hemocytes often bind to other thin trachea but not to larger trachea, as is evident in the comparative scarcity of hemocytes bound to the dorsal longitudinal tracheal trunks (Fig. 4a, b). A similar phenomenon also occurs in adult mosquitoes; though lacking tracheal tufts and their free-floating tracheoles, hemocytes in adults often bind to thin trachea, and only occasionally bind to the thick trachea, such as those that replace the tracheal tufts in the eighth abdominal segment (Additional file 3: Figure S2). In summary, larvae contain a dense population of hemocytes, called the tracheal tuft hemocytes, in a region of the body that is functionally analogous to the periostial regions of adults.

### Larval tracheal tuft hemocytes are phagocytically active

**Fig. 5:**
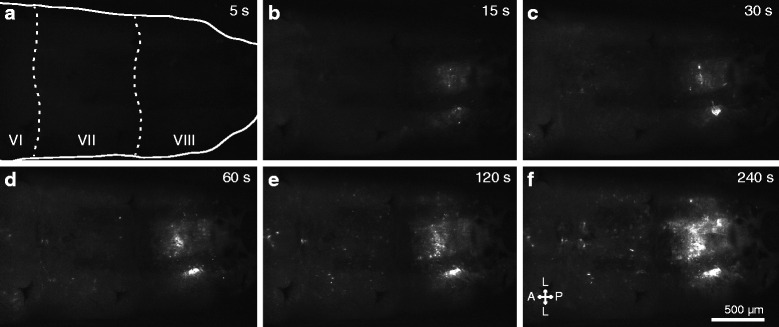
*E. coli* injected into the larval hemocoel rapidly aggregate in the tracheal tufts. **a–f** Still images from an intravital video (Additional file 4: Movie S2) recorded via fluorescence imaging through the posterior portion of the dorsal cuticle of a larva. GFP-*E. coli* (*white*) begin to accumulate at the tracheal tufts within 15 s after treatment. The still images shown were extracted at 5, 15, 30, 60, 120, and 240 s post-treatment. The *first panel* outlines the larva and marks abdominal segments 6–8 (VI–VIII). *Directional arrows*: *A* anterior, *P* posterior, *L* lateral

**Fig. 6:**
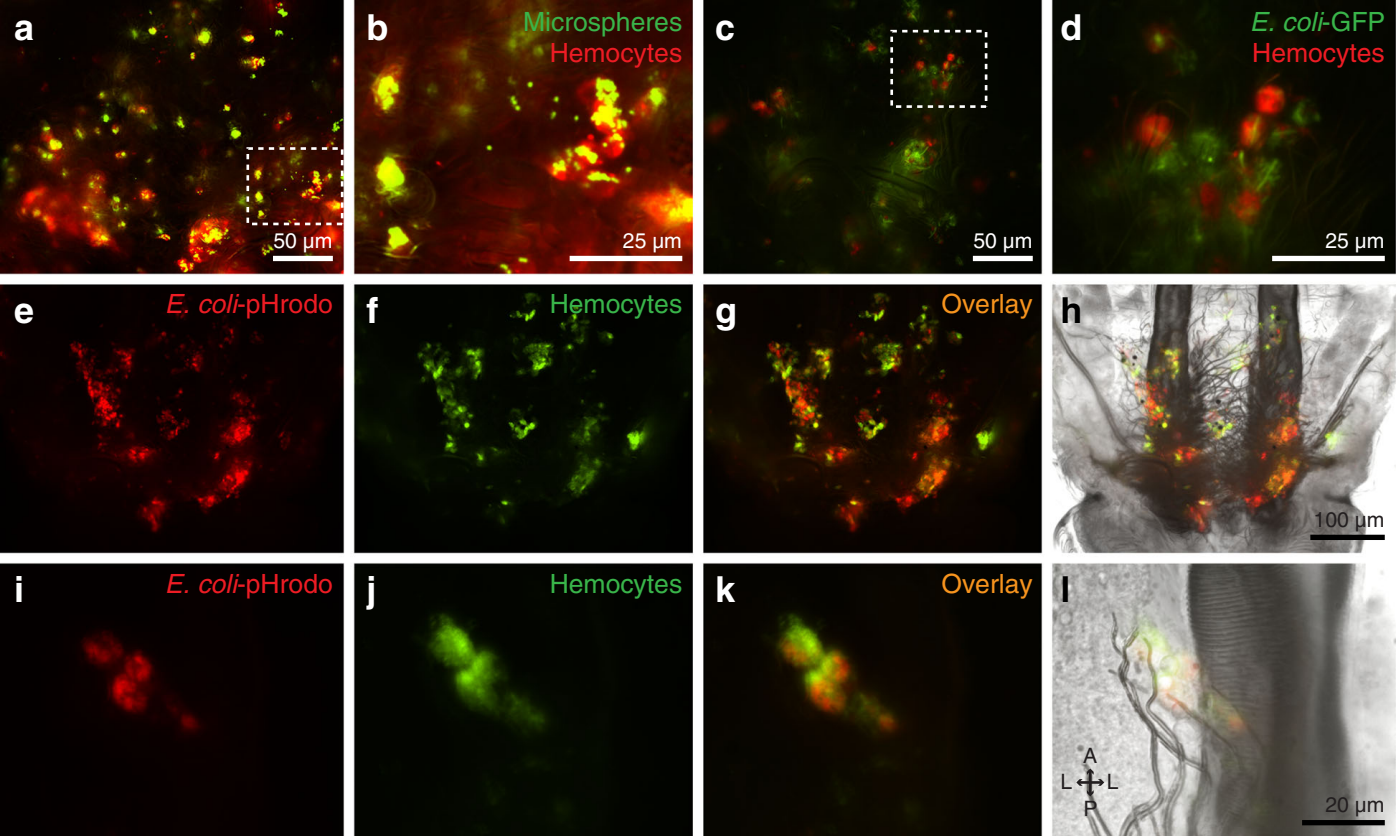
Tracheal tuft hemocytes show high phagocytic activity. **a–d** Fluorescence imaging of the phagocytic activity of tracheal tuft hemocytes (CM-DiI; *red*) at 4 h post-injection of 1 μm fluorescent microspheres (**a**, **b**) or GFP-*E. coli* (**c d**; *green*). *Rectangles* in **a** and **c** are magnified in **b** and **d**, respectively. **e–h** Series of fluorescence images (**e**–**g**) and bright-field overlay (**h**) of the tracheal tufts showing the significant overlap of pHrodo-*E. coli* fluorescence (*red*) and hemocyte fluorescence (DiO; *green*) at 4 h post-treatment. **i–l** Series of fluorescence images (**i–k**) and bright-field overlay (**l**) of a cluster of four tracheal tuft hemocytes (DiO; *green*) that have phagocytosed pHrodo-*E. coli* (*red*), showing a significant overlap in fluorescence signal. *Directional arrows*: *A* anterior, *P* posterior, *L* lateral


To test whether hemocytes engage in phagocytic immune responses while bound to the tracheal tufts, larvae were challenged with either green fluorescent microspheres, green fluorescent protein-tagged *Escherichia coli* (GFP-*E. coli*), or pHrodo-*E. coli*, and the challenge agent and the hemocytes (labeled in vivo with CM-DiI or CM-DiO) were imaged independently or in combination by fluorescence microscopy. Both microspheres and *E. coli* were rapidly phagocytosed by the tracheal tuft hemocytes, with bacterial aggregations at the tracheal tufts beginning within seconds after injection (Fig. 5; Fig. 6a–d; Additional file 4: Movie S2). Furthermore, when larvae were challenged with heat-killed *E. coli* bioparticles conjugated to pHrodo (a dye that only fluoresces in acidic environments such as those of a phagolysosome) and 4 h later the hemocytes were labeled, the pHrodo and hemocyte markers co-localized (Fig. 6e–l), further showing that the tracheal tuft hemocytes mount a rapid and robust phagocytosis response against bacteria that are flowing with the hemolymph.

**Additional file 4 Movie S2.** Phagocytosis of GFP-*E. coli* in the eighth segment tracheal tufts begins within seconds of infection. Four-minute time-lapse intravital video through the dorsal cuticle of a larva immediately following injection with GFP-*E. coli*, showing the rapid accumulation of fluorescent bacteria (*white*) in the eighth abdominal segment. GFP-*E. coli* begins to accumulate at the tracheal tufts in less than 15 s after treatment. Frame rate: 10 s of real time equals 1 s of movie.

### *E. coli* in the larval hemocoel aggregate in the tracheal tufts, where they are phagocytosed and killed

Injection of both fluorescent microspheres and GFP-*E. coli* into the hemocoel of larvae and adults resulted in patterns of aggregation that mirrored the respective hemocyte distributions in these two life stages: microspheres and *E. coli* aggregated at the periostial regions of the heart in adults and at the eighth abdominal segment tracheal tufts of larvae (Additional file 5: Figure S3 and Additional file 6: Figure S4). To quantitatively determine whether pathogen aggregation in larvae was indeed higher at the tracheal tufts when compared to the periostial regions of the heart, larvae were injected with GFP-*E. coli*, and the fluorescence emitted by these bacteria within the periostial regions and the tracheal tufts was measured at 4 and 24 h post-treatment (Additional file 7: Figure S5).

**Fig. 7:**
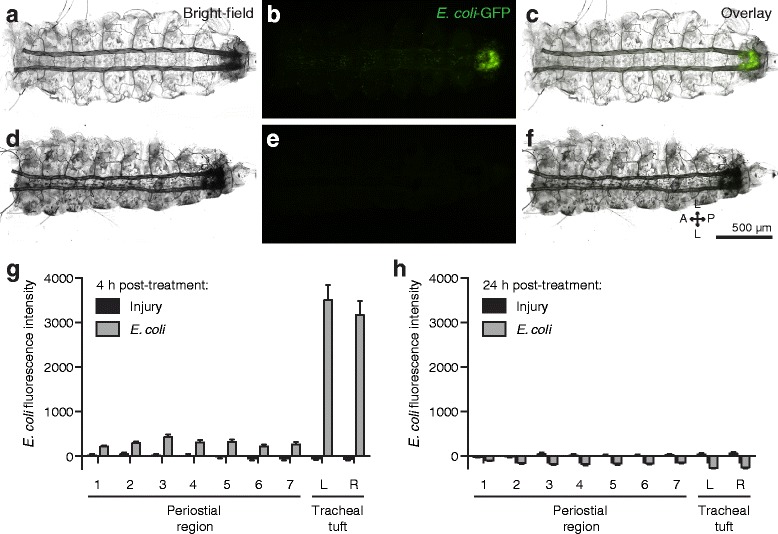
*E. coli* aggregate in the tracheal tufts, where they are rapidly destroyed. **a–f** Bright-field (**a**, **d**), fluorescence (**b**, **e**), and overlay (**c**, **f**) images of a dissected dorsal larval abdomen at 4 h (**a**–**c**) and 24 h (**d**–**f**) post-infection with GFP-*E. coli* (*green*). At 4 h, the bacteria have aggregated in the eighth abdominal segment, whereas at 24 h little or no fluorescence was observed. **g, h** Mean intensity of fluorescence signal in the periostial regions (1–7) and the left (*L*) and right (*R*) tracheal tufts of injured and GFP-*E. coli*-treated larvae at 4 h (**g**) and 24 h (**h**) post-treatment. At 4 h, mean intensities from both tracheal tufts of the *E. coli*-treated larvae were significantly higher than those from any region of interest (*ROI*) in injured larvae (*P* < 0.0001 for all comparisons). At 24 h, with the exception of periostial region 1 (*P* = 0.2990), mean intensities from each ROI were significantly lower in the *E. coli*-treated larvae compared to injured larvae (*P* ≤ 0.0005 for all other comparisons). In **g** and **h**, *whiskers* denote the standard error of the mean (*SEM*). For a graphical presentation of how the ROIs were constructed, see Additional file 7: Figure S5A. *Directional arrows*: A anterior, *P* posterior, *L* lateral

At 4 h post-treatment, GFP-*E. coli* formed distinct aggregates in the eighth abdominal segment of infected larvae, and this fluorescence signal was absent in injured (mock-injected) larvae (Fig. 7a–c, g; repeated measures two-way analysis of variance (RM 2 W ANOVA) *P* < 0.0001 for treatment). The strength of the fluorescence signal emitted by GFP-*E. coli*, which is indicative of their abundance, changed depending on the region of the body that was being measured (RM 2 W ANOVA *P* < 0.0001 for segment), and a Šidák multiple comparisons post hoc test showed that while there were no significant differences in the fluorescence signals between the different regions of injured larvae (*P* > 0.9999 for all comparisons), there were strong differences in infected larvae. Specifically, there were no significant differences between the GFP signals in the seven periostial regions of infected larvae (*P* ≥ 0.9926 for all comparisons), but the GFP signal from the left and right tracheal tufts was significantly higher than the GFP signal of any of the periostial regions (*P* < 0.0001 for all comparisons). Additional pairwise comparisons between injured and infected larvae showed that fluorescence in the periostial regions did not differ between infected and injured larvae (Šidák’s *P* ≥ 0.1069 for all seven comparisons), but that the fluorescence intensity in the tracheal tufts is significantly higher in the infected larvae when compared to injured larvae (Šidák’s *P* < 0.0001 for both comparisons). Finally, although some GFP fluorescence was detected in each of the periostial regions of infected larvae, upon closer examination this fluorescence was not due to pathogen aggregation in those areas but rather due to the uptake of GFP via the pinocytic activity of the pericardial cells and, to a lesser extent, from phagocytosis by the dorsal segmental hemocytes (Additional file 8: Figure S6). Together, these data show that, unlike in adults, bacteria do not aggregate in the periostial regions of larvae, which is as expected given that these regions are devoid of hemocytes. Instead, infection induces the massive aggregation of bacteria in the hemocyte-rich tracheal tufts.

At 24 h post-treatment, little or no GFP-*E. coli* was detected in any specimen, regardless of treatment (Fig. 7d–f, h). Interestingly, the fluorescence signal in infected larvae was significantly lower than that of the injured larvae (RM 2 W ANOVA *P* < 0.0001 for treatment), and a Šidák test that compared the different body regions of infected larvae to the corresponding body regions of injured larvae showed that, with the exception of the first periostial region (*P* = 0.2990), this was the case for all regions of the body (*P* ≤ 0.0005 for all other comparisons). Furthermore, there was an interaction between the type of treatment and the segment of the body being assayed (RM 2 W ANOVA *P* < 0.0001), indicating that the differences in the fluorescence signal emitted in different segments changed with treatment. Visual analysis of the data and a Šidák post hoc test showed that this is due to a more pronounced decrease in the fluorescence signal at the tracheal tufts relative to the periostial regions in infected larvae (*P* ≤ 0.0005 for all comparisons). Collectively, these findings indicate that larvae mount vigorous and effective antibacterial immune responses that are largely completed within 24 h of infection. These immune responses are strongest at the tracheal tufts, which surround the only incurrent openings of the larval dorsal vessel.

### Infection induces a robust melanization immune response at the tracheal tufts

The fluorescence signal emitted by GFP-*E. coli* at 4 h post-treatment is indicative of a vigorous phagocytosis response at the tracheal tufts, and the absence of this fluorescence at 24 h post-treatment is indicative of bacterial clearance. However, the fluorescence signal at 24 h is significantly lower than the fluorescence signal in injury controls, and visual examination of the specimens showed that antibacterial melanization deposits are minor at 4 h post-infection but far more prevalent 20 h later, with these deposits being most abundant at the tracheal tufts. We hypothesized that the negative fluorescence signal seen at 24 h after infection is due to the dampening of fluorescence by dark melanin deposits, and this hypothesis was supported by high magnification fluorescence imaging of the tracheal tufts of infected larvae (Additional file 9: Figure S7).

To quantitatively confirm that a melanization response is indeed taking place, we measured melanin deposition by calculating the mean pixel optical density (OD) in all the periostial regions as well as in the tracheal tufts of the same larvae used to calculate the phagocytosis response (see the preceding section; Additional file 7: Figure S5B). In this type of analysis, a higher OD value indicates darker pixels and therefore a stronger melanization response.

**Fig. 8:**
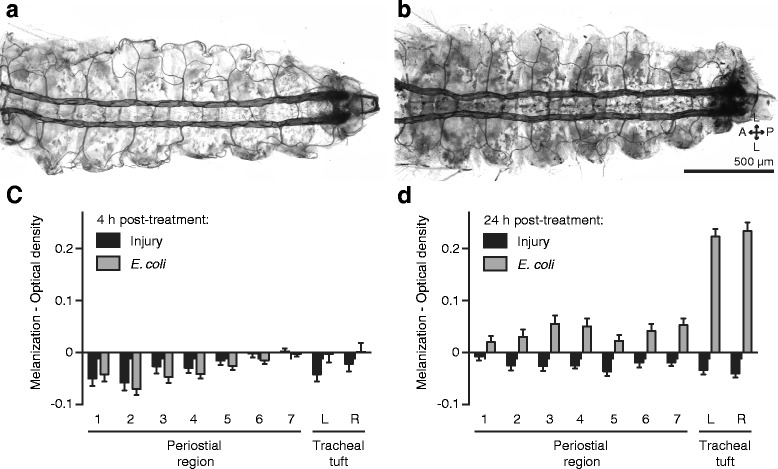
The melanization immune response is concentrated in the tracheal tufts. **a, b** Bright-field image of a dissected larval dorsal abdomen at 4 h (**a**) and 24 h (**b**) post-treatment with GFP-*E. coli*. At 4 h there is little or no visible melanization, whereas at 24 h widespread melanization is observed, including the uptake of melanin by the pericardial cells and an enrichment of melanin deposits in the tracheal tufts. **c, d** Melanization, as measured by mean pixel optical density (*OD*), in the periostial regions (1–7) and the left (*L*) and right (*R*) tracheal tufts of injured and GFP-*E. coli*-treated larvae at 4 h (**c**) and 24 h (**d**) post-treatment. At 4 h, OD values are similar in injured and *E*. coli-treated larvae (*P* = 0.9969), whereas at 24 h mean OD for all pairwise comparisons was significantly higher in infected larvae when compared to injured larvae (*P* ≤ 0.0079 for all other comparisons). In **c** and **d**, *whiskers* denote the SEM. For a graphical presentation of how the ROIs were constructed, see Additional file 7: Figure S5B. *Directional arrows*: *A* anterior, *P* posterior, *L* lateral

At 4 h post-treatment, little or no melanization was visible in either injured or *E. coli*-infected larvae (Fig. 8a, c; RM 2 W ANOVA *P* = 0.9969 for treatment). At 24 h post-treatment, however, melanization remained negligible in injured larvae yet increased dramatically in *E. coli*-infected larvae (Fig. 8b, d; RM 2 W ANOVA *P* < 0.0001 for treatment). With the exception of the first periostial region (Šidák’s *P* = 0.5972), the melanization response differed significantly across all pairwise comparisons between injured and infected larvae (Šidák’s *P* ≤ 0.0079 for all comparisons). Within the infected larvae, melanization was similar in all the periostial regions (Šidák’s *P* ≥ 0.1727 for all comparisons), but the melanization response in the tracheal tufts was significantly stronger than in any of the periostial regions (Šidák’s *P* < 0.0001 for all comparisons).

Because the periostial regions of larvae are devoid of hemocytes, we scrutinized the location of melanin deposits in these regions using a combination of fluorescence and bright-field microscopy, and found that melanin accumulates at the periostial regions of infected larvae because of the pinocytic activity of pericardial cells (Fig. 8b; Additional file 7: Figure S5B). At the tracheal tufts, however, melanin accumulation is much more pronounced, and this is primarily due to the accumulation and phagocytosis of melanized bacteria by the tracheal tuft hemocytes (Fig. 7; Fig. 8b, d; Additional file 9: Figure S7). Together, these data demonstrate a robust antibacterial melanization response in larvae, with much of the resultant melanin being deposited at the tracheal tufts, near the sole incurrent openings of the dorsal vessel.

### Hemocytes are more prevalent in the tracheal tufts but do not aggregate upon infection

**Fig. 9:**
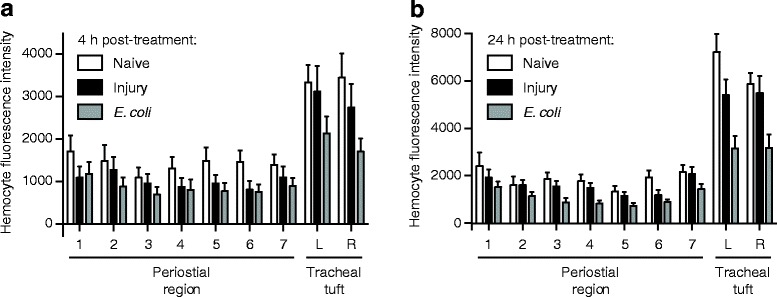
Hemocytes are more abundant in the tracheal tufts, but do not increase in response to *E. coli* infection. **a** Mean intensity of CM-DiI-stained hemocytes in the periostial regions (1–7) and the left (*L*) and right (*R*) tracheal tufts of naïve, injured, and GFP-*E. coli*-treated larvae at 4 h post-treatment. Hemocyte intensity was not significantly affected by treatment (RM 2 W ANOVA *P* = 0.1123) but was significantly higher in the tracheal tufts compared to the periostial regions in all three groups (Šidák’s *P* ≤ 0.0312 for 39 out of 42 comparisons). **b** Mean intensity of CM-DiI stained hemocytes in the periostial regions and tracheal tufts of naïve, injured, and GFP-*E. coli*-treated larvae at 24 h post-treatment. Hemocyte intensity was significantly affected by treatment (RM 2 W ANOVA *P* = 0.0012), with hemocyte intensities from GFP-*E. coli*-treated larvae being significantly lower than those of both naïve and injured larvae (Šidák’s *P* < 0.0001 for all comparisons). Hemocyte intensity was significantly higher in the tracheal tufts compared to the periostial regions in all three groups (Šidák’s *P* ≤ 0.0003 for all comparisons). *Whiskers* denote the SEM. For a graphical presentation of how the ROIs were constructed, see Additional file 7: Figure S5C

In adult mosquitoes, infection induces the migration of circulating hemocytes, whereby many exit circulation and bind to the initial population of sessile hemocytes present at the periostial regions of the heart [9, 13]. To test whether pathogen accumulation in the eighth abdominal segment tracheal tufts of larvae is accompanied by further hemocyte aggregation in this region, naïve, injured and *E. coli*-infected larvae were incubated for 4 or 24 h, the hemocytes were stained in vivo with CM-DiI, and the fluorescence signal emitted by the hemocytes was measured (Fig. 9; Additional file 7: Figure S5C).

At 4 h post-treatment, there was no difference in the fluorescence signal emitted by hemocytes in naïve, injured, and infected mosquitoes (Fig. 9a; RM 2 W ANOVA *P* = 0.1123 for treatment). However, there was a significant difference between the regions of the body being measured (RM 2 W ANOVA *P* < 0.0001). Specifically, for naïve, injured, and infected mosquitoes, the fluorescence intensities of the two tracheal tufts were significantly higher than the fluorescence intensities in the corresponding periostial regions (Šidák’s *P* ≤ 0.0312 for 39 out of 42 comparisons). This indicates that hemocytes are absent from the periostial regions of infected larvae (any signal emanates from segmental hemocytes) but are abundant at the tracheal tufts. This also indicates that at 4 h post-infection the relative number of tracheal tuft hemocytes in infected larvae is similar to that found in naïve and injured larvae.

At 24 h post-treatment, hemocyte intensity was significantly different between naïve, injured, and infected larvae (Fig. 9b; RM 2 W ANOVA *P* = 0.0012). There was also a significant difference between the regions of the body being measured (RM 2 W ANOVA *P* < 0.0001), as the fluorescence signal in the tracheal tufts remained elevated relative to the periostial regions (Šidák’s *P* ≤ 0.0003 for all comparisons). Furthermore, there was a significant interaction between the region of the body and the treatment (RM 2 W ANOVA *P* < 0.0001 for interaction), indicating that the distribution of fluorescence signal in the different body regions changes depending on the infection status of the larva. Specifically, the fluorescence signal emitted by segmental hemocytes was similar between the periostial regions of interest (ROIs) of naïve, injured, and infected larvae (Šidák’s *P* ≥ 0.1528 for all comparisons), but the signal in the tracheal tufts was lower in infected larvae when compared to naïve and injured larvae (Šidák’s *P* < 0.0001 for all comparisons).

Taken together, these results suggest that although hemocytes are more abundant at the tracheal tufts, infection does not recruit circulating hemocytes to the periostial regions or to the tracheal tufts. At 24 h post-treatment, hemocyte fluorescence in the tracheal tufts of infected larvae was lower than that of naïve and injured larvae, but visual analysis of these specimens strongly suggests that this is not due to a reduction in the hemocyte population but rather due to melanization-induced dampening of the fluorescence signal (Fig. 7h; Fig. 8d; Additional file 7: Figure S5B and Additional file 9: Figure S7).

## Discussion

**Fig. 10:**
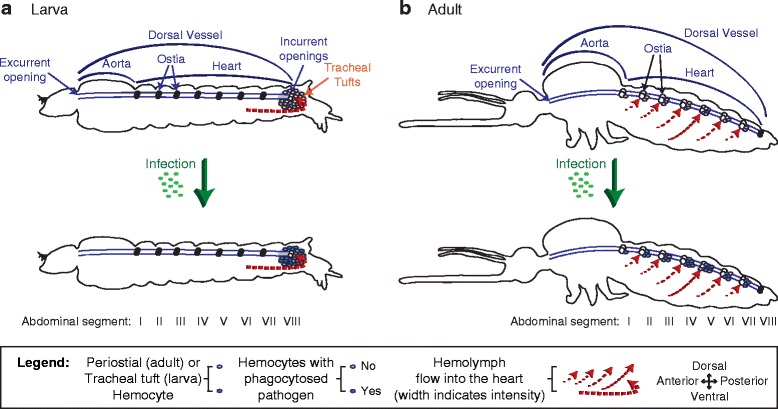
The circulatory and immune systems of mosquito larvae and adults are functionally integrated. **a** In larvae, hemolymph enters the heart via the posterior incurrent openings of the heart. At this location, tracheal tuft hemocytes phagocytose and kill pathogens as they flow with the hemolymph while en route to the heart. Infection does not induce the migration of hemocytes to the tracheal tufts. **b** In adults, hemolymph enters the heart via ostia located in abdominal segments 2–7. At these locations, periostial hemocytes phagocytose and kill pathogens as they flow with the hemolymph while en route to the heart. Infection induces the migration of additional hemocytes to the periostial regions, where they amplify the phagocytosis response. Illustrations are not drawn to scale

Mosquitoes undergo a dramatic metamorphosis as they transition from an aquatic environment during the larval and pupal stages to terrestrial and aerial environments during the adult stage. In the present study, we show that the changes in circulatory and respiratory physiology that occur during the larval-to-adult transition are accompanied by stage-specific interactions between the circulatory, immune, and respiratory systems (Fig. 10). These systems are functionally integrated such that structures once thought to act solely in oxygen dissemination have been co-opted by the immune system to act as both a sieve to sequester circulating pathogens and as an ideal staging ground for mounting cellular and humoral immune responses at the sole entry points for hemolymph into the heart.

A major finding in this study is that bacteria aggregate and are killed by hemocytes that are attached to the tracheal tufts of mosquito larvae, which are structures that surround the only incurrent openings of the heart. This represents an immune strategy where pathogen sequestration and killing occurs in the areas of the body that receive the highest amount of hemolymph flow. These locations are ideal for mounting immune responses, as they increase the probability of hemocytes encountering circulating pathogens and facilitate the rapid dissemination of hemocyte-produced humoral immune factors. Immune responses at the tracheal tufts are functionally, though not spatially, analogous to the immune responses that occur in the periostial regions of adult mosquitoes [9, 12, 13], as in both life stages pathogens and hemocytes associate with the entry points for hemolymph into the heart. Similar aggregations of pathogens at the periostial regions have also been observed in adult *Drosophila*, though these aggregations have not been linked to hemolymph flow or the structural mechanics of the heart, and have not been scrutinized at the cellular level [23, 28]. Furthermore, although melanotic nodules and melanized bacteria accumulate on the surface of the heart of lepidopteran larvae [29, 30], these melanin deposits are diffused along the length of the heart and do not associate specifically with the periostial regions (the tracheal tufts were not examined). Thus, it remains unclear whether these melanization responses in larval Lepidoptera are modulated by hemolymph flow.

A difference between the immune response at the periostial regions of adult mosquitoes and the immune response at the tracheal tufts of larvae is that infection induces the migration of additional hemocytes to the former but not the latter. The reason for this is unclear, but it may be due to the kinetics of the antibacterial immune response in these two life stages. In adults, we have observed bacteria at the periostial regions as far as 12 days post-infection (the last time point that was studied) [9], and other studies have documented the presence of bacteria in the hemocoel days or weeks after infection [31, 32]. In larvae, however, our method measuring fluorescence pixel intensity could not detect any bacteria at 24 h post-infection, and higher magnification observation of larval tissues detected few bacteria. Another possibility for the lack of an infection-induced increase in the number of hemocytes in the tracheal tufts is that, unlike in adults, larval hemocytes do not increase in number following immune stimulation or alter their adherence properties upon infection, as occurs in adults after blood feeding or immune stimulation [9, 12, 13, 33–36]. However, as these properties have yet to be tested in larval hemocytes, we presently hypothesize that the basal number of hemocytes at the tracheal tufts, in combination with the activity of other sessile and circulating hemocytes, is sufficient to rapidly clear systemic infections. This immune proficiency may in part be facilitated by the streamlined circulatory system of larvae, which contains a single pair of incurrent heart openings [11], as opposed to that of adults, which contains seven pairs of incurrent ostia [10].

In adult mosquitoes, the primary cause of pathogen death at the periostial regions is phagocytosis [9, 13], and our data show that this immune response is also prominent at the tracheal tufts of mosquito larvae. This response begins within seconds of infection, which is similar to the speed of the phagocytosis response by the periostial, non-cardiac sessile, and circulating hemocytes of adult mosquitoes [4, 9, 13, 37–40]. Furthermore, much like in adult mosquitoes [2, 9, 38, 39], melanization of bacteria is prevalent in larvae. Our observation that melanin deposits are predominant at the tracheal tufts suggests that hemolymph flow drives the accumulation of melanized bacteria at these respiratory structures, and some of the melanized bacteria are phagocytosed by hemocytes. These observations also provide a functional explanation for the spatial distribution of the melanization response against vertically transmitted *Elizabethkingia meningoseptica* that has been observed in *A. gambiae* larvae [41], which also occurs in the eighth abdominal segment. Furthermore, our findings provide a new host-immunity perspective on a previous report linking parasitoid wasp development to the tracheal tufts of *Heliothis virescens* caterpillars, where the authors suggest that the trapping of *Toxoneuron nigriceps* eggs in the tracheal tufts is advantageous for the development of the parasite because it places them in an environment that is rich in oxygen [42]. Although the authors note that the propensity of the eggs to aggregate near the tracheal tufts is due to the natural flow of hemolymph, it is possible that the host immune response at the tracheal tufts outweighs any respiration-related benefits gained by the wasp eggs.

Our discovery that a high concentration of immunologically active hemocytes is associated with the eighth abdominal segment tracheal tufts of *A. gambiae* larvae represents the first description of this unique population of sessile hemocytes in Diptera. Populations of hemocytes in the posterior abdominal segments have been observed in other dipteran larvae, such as *Drosophila* [43–52], *Calliphora* sp. [53–55], and *Musca* sp. [53, 56]. However, unlike the tracheal tuft-bound sessile hemocytes we describe in mosquito larvae, the posterior hemocytes described in other Diptera have not been documented as a hot spot of phagocytic activity during systemic infections or described as being associated with tracheal tufts.

Mosquito larvae are metapneustic, meaning they breathe through a single pair of posterior spiracles [57]. Air enters these spiracles and is transported the length of the body by a pair of large dorsal longitudinal tracheal trunks. As they traverse the body, these tracheal trunks branch into a series of smaller segmental branches, which branch into increasingly smaller trachea, finally terminating as thin-walled tracheoles that directly oxygenate the tissues to which they are permanently attached. However, the tracheoles that comprise the eighth abdominal segment tracheal tufts differ from other tracheoles in that, aside from some attachments to the posterior terminus of the heart, they float freely in the hemolymph instead of being attached to tissues [11].

Tracheal tufts, which have also been referred to as the “tracheole plexus” or “tracheal lungs,” have been observed in the larvae of the mosquitoes *Aedes aegypti* [58], *Culex* sp. [59, 60], *Anopheles maculipennis* [59, 61], and *Anopheles quadrimaculatus* [62], as well as in the larvae of other dipterans [59, 63, 64], but not in *Drosophila*, as our own experiments have confirmed. However, the tracheal tufts have been most comprehensively described in the lepidopteran *Calpodes ethlius* [65]. Because of their obvious role in the oxygenation of hemolymph and their close association with hemocytes, the tracheal tufts were originally hypothesized to act as a “lung” for hemocytes. However, given that circulating and sessile hemocytes persist following eclosion and the tracheal tufts do not, we hypothesize that the tracheal tufts are not necessary for the direct oxygenation of hemocytes and instead function in the dissolution of oxygen into the hemolymph. This is supported by our observation that hemolymph flow is highest at the tracheal tufts [11], and thus, the contractile action of the heart disseminates newly dissolved oxygen to other parts of the body. More relevant to immune defense, we hypothesize that hemocytes associate with the tracheal tufts because, in a process analogous to the function of periostial hemocytes [9, 12, 13], the attachment of immune cells to structures that surround the only incurrent opening of the heart places them in an ideal location to survey the hemolymph for foreign invaders. Additionally, we observed that hemocytes, both in larvae and adults, have a propensity for binding to thin trachea, and we hypothesize that this also confers an immunological advantage, because the sites for gas exchange are also locations where pathogens often invade the hemocoel [12, 66–73].

To our knowledge, this study contains the first description of segmentally arranged sessile hemocytes in larval mosquitoes. These segmentally arranged cells are phagocytic, but infection does not induce the aggregation of hemocytes or the dense aggregation of pathogens within these locations. Because of their immune competence and their sheer abundance in the larval stage, this newly discovered population of sessile hemocytes represents a formidable immune reservoir. A similar arrangement of hemocytes, referred to as the segmentally arranged sessile hemocyte islets or patches, has been documented in *Drosophila* [44, 46, 47, 50, 52, 74, 75], as well as in other Diptera, such as the larger *Calliphora* flies [54]. In *Drosophila*, larval segmental hemocytes detach at the onset of pupation to engage in tissue remodeling [46, 50, 76] and in order to encapsulate parasitoid wasp eggs [48, 52]. In adult mosquitoes, segmentally arranged hemocytes participate in the consumption of autolysing larval swim muscles during the first couple of days post-eclosion, and then dissipate [12]. Our finding that these cells also contribute to bacterial clearance points to an even broader role for these sessile hemocytes in larval insect immune responses.

## Conclusions

*A. gambiae* is a major vector of malaria in sub-Saharan Africa, and as such, understanding the innate immune defenses of this mosquito against invading pathogens is crucial to the development of novel pest and disease control programs. In spite of this, the host immune response in larvae has received scant attention compared to the adult stage, a significant oversight considering that larvae are often a major target in pest control efforts [77, 78], and many common bacterial and fungal pesticides are seeded into mosquito breeding sites where they exert their effects in the hemocoel of larvae [79–82]. The present study uncovers an immune strategy that mosquito larvae use in combating microbial infections, which relies on the coordinated action of their circulatory, immune, and respiratory systems to sequester and kill pathogens present in the hemocoel.

## Methods

### Mosquito rearing and maintenance

*Anopheles gambiae* Giles *sensu stricto* (G3 strain; Diptera: Culicidae) were reared as previously described [83]. Briefly, larvae were reared in deionized water and fed a mixture of koi food and baker’s yeast. Upon pupation, mosquitoes were transferred to plastic containers with a marquisette net top, and adults were fed a solution of 10 % sucrose *ad libitum*. All life stages were maintained in an environmental chamber set to 27 °C and 75 % relative humidity under a 12 h:12 h, light:dark cycle. Experiments were conducted on fourth instar larvae and adult female mosquitoes at 5 days post-eclosion.

### Mosquito injection, inoculation of immune elicitors, and bacterial infection

Mosquito larvae were immobilized by removing excess water and then injected at the lateral center of the mesothorax. Mosquito adults were cold anesthetized and then injected at the thoracic anepisternal cleft. Finely pulled glass capillary tubes were used as needles, and 0.2 μl of the following substances, alone or in combination, were injected: 0.08 % solids 1-μm diameter green fluorescent (505/515) carboxylate-modified microspheres (Invitrogen, Carlsbad, CA, USA) in phosphate-buffered saline (PBS) (pH 7.0), GFP-expressing *E. coli* (modified DH5α) in Luria-Bertani’s rich nutrient medium (LB), LB medium, 1 mg/ml pHrodo Red *E. coli* Bio Particles (560/585 nm; Invitrogen) in PBS, or cellular staining solutions (see the next section). For bacterial infections, *E. coli* were grown in LB overnight at 37 °C in a shaking incubator until cultures reached approximately OD_600_ = 5, as measured using a BioPhotometer Plus spectrophotometer (Eppendorf AG, Hamburg, Germany).

### Staining of hemocytes, pericardial cells, and heart muscle

To fluorescently label hemocytes, live larvae or adults were injected with 67 μM Vybrant CM-DiI or DiO Cell-Labeling Solutions (Invitrogen) in PBS and were incubated at 27 °C and 75 % relative humidity for 20 min or 1 h, respectively. Larvae were then fixed in 16 % paraformaldehyde (Electron Microscopy Sciences, Hatfield, PA, USA) for 1 h and either rinsed briefly in PBS and the whole body mounted directly on a glass slide using Aqua-Poly/Mount (Polysciences Inc., Warrington, PA, USA), or dissected along a coronal plane, rinsed briefly in PBS, and the dorsal abdomen sans the internal organs mounted. Adults were fixed by injecting 16 % paraformaldehyde, and 10 min later the specimens were dissected along a coronal plane, placed in 0.5 % Tween in PBS to reduce surface tension, rinsed briefly in PBS, and the dorsal abdomen sans the internal organs mounted on a glass slide using Aqua-Poly/Mount.

To fluorescently co-stain phagocytic events and hemocytes, larvae were injected with green fluorescent microspheres in PBS, GFP-*E. coli* in LB, or pHrodo Red *E. coli* Bio Particles in PBS and allowed to incubate for 4 h at 27 °C and 75 % relative humidity prior to staining with either CM-DiI or DiO. To co-stain hemocytes and heart muscle, following staining with CM-DiI the larvae were incubated in 16 % formaldehyde for 1 h, dissected, and incubated in 0.7 μM phalloidin-Alexa Fluor 488 (Invitrogen) in PBS on a Pelco R1 Rotary Mixer (Pelco Instruments, Redding, CA, USA) for 1 h. To fluorescently stain pericardial cells, larvae and adults were injected with 0.2 mg/ml Alexa Fluor conjugated IgG 568 nm (Invitrogen) and 0.8 mM Hoechst 33342 nuclear stain (350/461; Invitrogen) in PBS and allowed to incubate for 2 h. To co-stain pericardial cells and hemocytes, larvae were injected with 0.2 mg/ml IgG 488 nm and allowed to incubate for 2 h prior to staining hemocytes with CM-DiI. After staining, specimens were dissected and mounted as described above.

### Light and fluorescence microscopy of aldehyde-fixed mosquito specimens

Larval and adult specimens were imaged under bright-field and fluorescence illumination using a Nikon 90i compound microscope connected to a Nikon Digital Sight DS-Qi1Mc monochrome digital camera (Nikon, Tokyo, Japan). For the rendering of detailed fluorescence images with extended focal depth, Z-stacks of whole mounts were acquired using a linear encoded Z-motor, and all images in a stack were combined to form a single focused image using the Extended Depth of Focus (EDF; for image viewing) or Maximum Intensity Projection (for image quantification) modules of Nikon’s NIS Elements software. For three-dimensional rendering, Z-stacks were quantitatively deconvolved using the AQ 3D Blind Deconvolution module of NIS Elements and rendered using the volume view feature.

### Quantification of fluorescently labeled *E. coli* and hemocyte fluorescence intensity

GFP-*E. coli* and CM-DiI-labeled hemocyte fluorescence intensity were quantified in abdominal segments 1–8 of larvae using maximum intensity projections of Z-stacks created in NIS Elements software. For abdominal segments 1–7, custom polygonal regions of interest (ROIs) were drawn over the anterior portion of each abdominal segment, encompassing the periostial regions of the heart (Additional file 7: Figure S5). Specifically, these ROIs contained the area in each abdominal segment that lay between the dorsal longitudinal tracheal trunks, and stretched from the abdominal suture to the dorsal abdominal tracheal commissure. For segment 8, two custom ROIs circumscribing each tracheal tuft were constructed. The mean pixel intensity, defined as the average intensity of pixels within a given ROI, was then calculated to compare fluorescence emission across ROIs quantitatively.

For GFP-*E. coli* quantification, intensity measurements for any given ROI in the GFP-*E. coli* and injury (injected with LB alone) groups were normalized by subtracting the intensity values from the corresponding ROI from the naïve group, as naïve mosquitoes represent background fluorescence. Three independent trials were conducted at both 4 and 24 h post-treatment, and each trial consisted of at least 10 mosquitoes per group. For CM-DiI-labeled hemocyte quantification, intensity measurements for any given ROI in the CM-DiI-stained naïve, injured, and *E. coli* groups were normalized by subtracting the intensity values from the corresponding ROI of the unstained naïve group. Four and five independent trials consisting of on average 8 and 6 mosquitoes per group were conducted at 4 and 24 h post-treatment, respectively. Because for each mosquito measurements were obtained for all periostial regions and the tracheal tufts (left and right), data (Additional file 10: Data file S1) were analyzed by repeated measures two-way ANOVA (RM 2 W ANOVA; repeated measures being the nine regions of the body of an individual mosquito), using treatment and ROI as the variables, followed by a Šidák *post hoc* test.

### Quantification of melanization in response to *E. coli* infection

Melanization was quantified in abdominal segments 1–8 of larvae using maximum intensity projections created from the bright-field images of the Z-stacks of the same specimens used to measure GFP-*E. coli* fluorescence intensity. These data (Additional file 10: Data file S1) were analyzed using the same ROI scheme and statistical tests described above. For the melanization analysis, the mean optical pixel density (OD), defined as the average OD of pixels within a given ROI, was calculated to compare melanization across ROIs quantitatively. The OD measurements for any given ROI in the *E. coli* and injured groups were normalized by subtracting the OD values from the corresponding ROI from the naïve group.

## Additional files

Additional file 1: Figure S1. Tracheal tufts are shed in the fourth instar larval exuviae during pupation. (**A**) Bright-field image of a lateral view of fourth instar larval exuviae showing that the eighth abdominal segment trachea (*black rectangle*), including the tracheal trunks (*T*) and the tracheal tufts (*TT*), are shed during pupation. (**B**) Bright-field image of a dissected pupal dorsal abdomen, showing the dorsal longitudinal tracheal trunks (*T*) and the absence of the eighth abdominal segment tracheal tufts (*circle*). *Directional arrows*: *A* anterior, *P* posterior, *D* dorsal, *V* ventral, *L* lateral. (PDF 391 kb) Additional file 3: Figure S2. The sessile hemocytes of adults preferentially bind thin trachea. (**A, B**) Bright-field overlay (**A**) and fluorescence (**B**) images showing hemocytes (CM-DiI; *red*) bound to thin abdominal trachea. (**C, D**) Bright-field overlay (**C**) and fluorescence (**D**) images showing very few hemocytes bound to the large trachea of the eighth abdominal segment, which lacks tracheal tufts. *Directional arrows*: *A* anterior, *P* posterior, *L* lateral. (PDF 404 kb) Additional file 5: Figure S3. Microspheres injected into larvae and adults aggregate in regions of high hemolymph flow and hemocyte concentration. (**A–F**) Dissected larval (**A**–**C**) and adult (**D**–**F**) dorsal abdomens with fluorescently labeled hemocytes (CM-DiI; *red*) at 4 h after injection with fluorescent microspheres (*green*). In larvae, microspheres preferentially aggregated in the eighth abdominal segment (**A**), where there is a high concentration of hemocytes (**B**, *circle* in **C**). In adults, microspheres preferentially aggregated at the periostial regions of the heart (**D**), where the periostial hemocytes are located (**E**, *arrows* in **F**). (**G–L**) Dissected larval (**G**–**I**) and adult (**J**–**L**) dorsal abdomens with fluorescently labeled hemocytes at 24 h after injection with fluorescent microspheres, showing an aggregation pattern identical to that observed at 4 h post-treatment. *Directional arrows*: *A* anterior, *P* posterior, *L* lateral. (PDF 1117 kb) Additional file 6: Figure S4.
*E. coli* injected into larvae and adults aggregate in regions of high hemolymph flow and hemocyte concentration. (**A–F**) Dissected larval (**A**–**C**) and adult (**D**–**F**) dorsal abdomens with fluorescently labeled hemocytes (CM-DiI; *red*) at 4 h after injection with GFP-*E. coli* (*green*). In larvae, *E. coli* preferentially aggregated in the eighth abdominal segment (**A**), where there is a high concentration of hemocytes (**B**, *circles* in **C**). In adults, *E. coli* aggregated at the periostial regions of the heart (**D**), where the periostial hemocytes are located (**E**, *arrows* in **F**). (**G–L**) Dissected larval (**G**–**I**) and adult (**J**–**L**) dorsal abdomens with fluorescently labeled hemocytes at 24 h after injection with GFP-*E. coli*. The aggregation pattern of *E. coli* in adults at 24 h after treatment was similar to that observed at 4 h post-treatment, but in larvae, fluorescence from *E. coli* was not observed anywhere in the body because the infection had been largely cleared. *Diagonal lines* in panels **G**–**I** denote the edges of rotated images. *Directional arrows*: *A* anterior, *P* posterior, *L* lateral. (PDF 679 kb) Additional file 7: Figure S5. Fluorescence intensity and optical density at the larval periostial regions and tracheal tufts were measured using custom-drawn regions of interest. (**A–C**) Representative fluorescence and bright-field images of dissected dorsal abdomens from larvae infected with GFP-*E. coli* for 4 h (**A**) and 24 h (**B**), and from a naïve larva (**C**). Images such as these were used to quantify mean fluorescence intensity of GFP-*E. coli* (**A**) and CM-DiI-stained hemocytes (**C**), as well as the mean optical density (*OD*) of melanin deposits (**B**). Custom regions of interest (*ROIs*) 1–7 contain each periostial region (*PR*), delineated as the area in each abdominal segment that lies between the dorsal longitudinal tracheal trunks and stretches from the abdominal suture to the dorsal abdominal tracheal commissure. ROIs 8 and 9 contain the left and right tracheal tufts (*TT*). *Directional arrows*: *A* anterior, *P* posterior, *L* lateral. (PDF 449 kb) Additional file 8: Figure S6. GFP signal from periostial region ROIs of larvae originate from pericardial cells and segmental hemocytes. (**A–C**) Fluorescence images taken at 4 h post-treatment showing that pericardial cells (**A**; IgG 568 nm, *red*) pinocytose GFP (**B**) that is flowing with the hemolymph following its release from degrading GFP-*E. coli* (overlay of **A** and **B** is shown in **C**). (**D–F**) Bright-field and fluorescence images of a portion of the seventh and eighth abdominal segments of a dissected larva at 4 h post-injection with GFP-*E. coli* (*green*), showing that both the tracheal tuft hemocytes and the dorsal segmental hemocytes (CM-DiI; *red*) engage in phagocytosis. A group of segmental hemocytes (**D**, **E**, *rectangle*) that have phagocytosed *E. coli* within the seventh abdominal segment region of interest (area within *dashed outline*) is magnified in **F**. Neither pathogens nor hemocytes were detected within the periostial regions. *Directional arrows*: *A* anterior, *P* posterior, *L* lateral. (PDF 471 kb) Additional file 9: Figure S7. Tracheal tuft hemocytes phagocytose melanized bacteria. (**A, B**) Fluorescence (**A**) and bright-field overlay (**B**) images of tracheal tuft hemocytes (CM-DiI; *red*) phagocytosing aggregates of melanized (*black*) GFP-*E. coli* (*green*) at 4 h post-infection. Notice that melanin deposits dampen the fluorescence signal. *Directional arrows*: *A* anterior, *P* posterior, L lateral. (PDF 232 kb) Additional file 10: Data file S1. Fluorescence and optical density (*OD*) data. (XLSX 91 kb)
