# Supplementary material for: Functional integration of the circulatory, immune, and respiratory systems in mosquito larvae: pathogen killing in the hemocyte-rich tracheal tufts
Source: BMC Biol. 2016 Sep 19;14:78. doi: 10.1186/s12915-016-0305-y (PMC5027632; doi:10.1186/s12915-016-0305-y)
Supplement: Additional file 1: Figure S1. — Tracheal tufts are shed in the fourth instar larval exuviae during pupation. (A) Bright-field image of a lateral view of fourth instar larval exuviae showing that the eighth abdominal segment trachea (black rectangle), including the tracheal trunks (T) and the tracheal tufts (TT), are shed during pupation. (B) Bright-field image of a dissected pupal dorsal abdomen, showing the dorsal longitudinal tracheal trunks (T) and the absence of the eighth abdominal segment tracheal tufts (circle). Directional arrows: A anterior, P posterior, D dorsal, V ventral, L lateral. (PDF 391 kb) [file 12915_2016_305_MOESM1_ESM.pdf]

Functional integration of the circulatory, immune, and respiratory systems in mosquito larvae: pathogen killing in the hemocyte-rich tracheal tufts

Garrett P. League and Julián F. Hillyer (julian.hillyer@vanderbilt.edu)

Department of Biological Sciences, Vanderbilt University, Nashville, TN, U.S.A.

BMC Biology, 2016

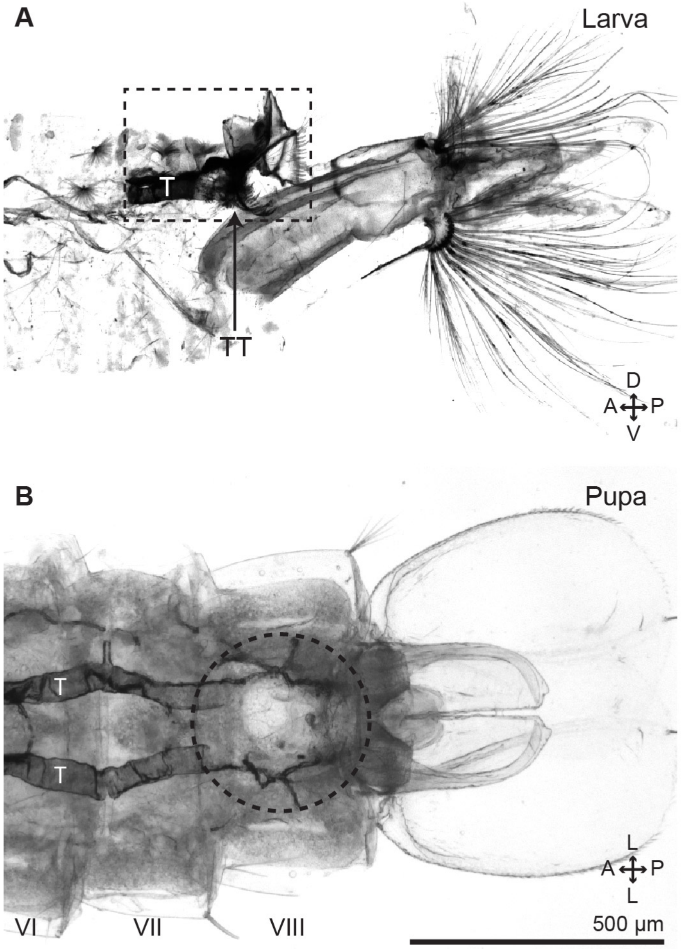

Additional File 1: Figure S1. Tracheal tufts are shed in the 4th instar larval exuvium during pupation. (**A**) Bright-field image of a lateral view of a 4th instar larval exuvium showing that the 8th abdominal segment trachea (black rectangle), including the tracheal trunks (T) and the tracheal tufts (TT), are shed during pupation. (**B**) Bright-field image of a dissected pupal dorsal abdomen, showing the dorsal longitudinal tracheal trunks (T) and the absence of the 8th abdominal segment tracheal tufts (circle). Directional arrows: A, anterior; P, posterior; D, dorsal; V, ventral; L, lateral.
