# Supplementary material for: Functional integration of the circulatory, immune, and respiratory systems in mosquito larvae: pathogen killing in the hemocyte-rich tracheal tufts
Source: BMC Biol. 2016 Sep 19;14:78. doi: 10.1186/s12915-016-0305-y (PMC5027632; doi:10.1186/s12915-016-0305-y)
Supplement: Additional file 3: Figure S2. — The sessile hemocytes of adults preferentially bind thin trachea. (A, B) Bright-field overlay (A) and fluorescence (B) images showing hemocytes (CM-DiI; red) bound to thin abdominal trachea. (C, D) Bright-field overlay (C) and fluorescence (D) images showing very few hemocytes bound to the large trachea of the eighth abdominal segment, which lacks tracheal tufts. Directional arrows: A anterior, P posterior, L lateral. (PDF 404 kb) [file 12915_2016_305_MOESM3_ESM.pdf]

Functional integration of the circulatory, immune, and respiratory systems in mosquito larvae: pathogen killing in the hemocyte-rich tracheal tufts

Garrett P. League and Julián F. Hillyer (julian.hillyer@vanderbilt.edu)

Department of Biological Sciences, Vanderbilt University, Nashville, TN, U.S.A.

BMC Biology, 2016

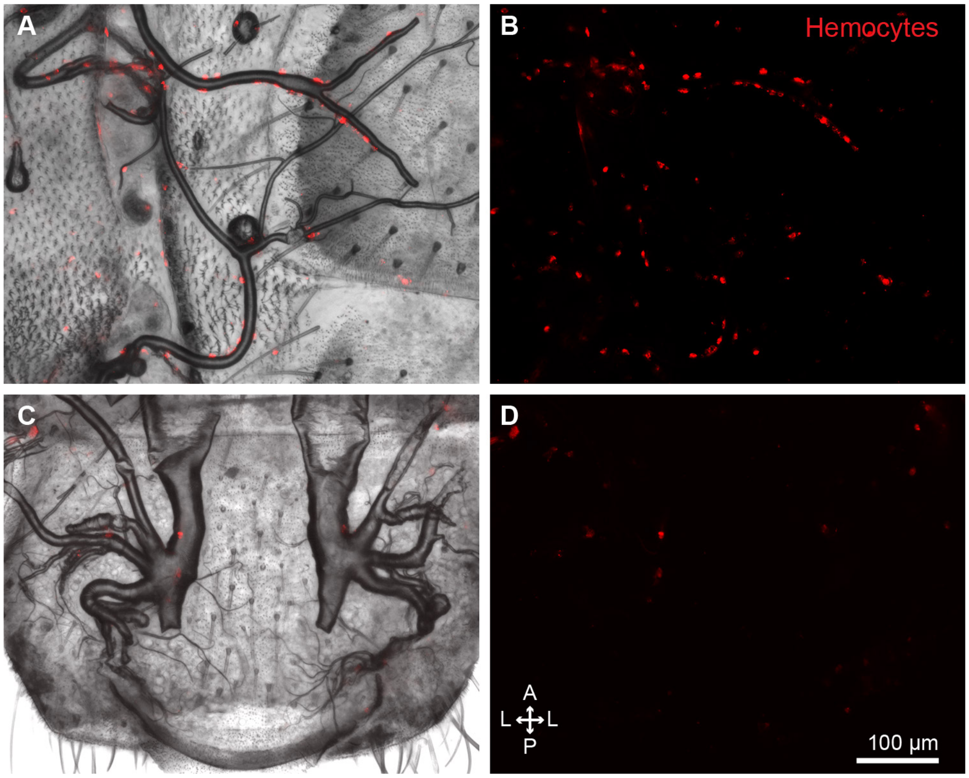

Additional File 3: Figure S2. The sessile hemocytes of adults preferentially bind thin trachea. (**A-B**) Bright-field overlay (A) and fluorescence (B) images showing hemocytes (CM-DiI; red) bound to thin abdominal trachea. (**C-D**) Bright-field overlay (C) and fluorescence (D) images showing very few hemocytes bound to the large trachea of the 8th abdominal segment, which lacks tracheal tufts. Directional arrows: A, anterior; P, posterior; L, lateral.
