# Supplementary material for: Functional integration of the circulatory, immune, and respiratory systems in mosquito larvae: pathogen killing in the hemocyte-rich tracheal tufts
Source: BMC Biol. 2016 Sep 19;14:78. doi: 10.1186/s12915-016-0305-y (PMC5027632; doi:10.1186/s12915-016-0305-y)
Supplement: Additional file 5: Figure S3. — Microspheres injected into larvae and adults aggregate in regions of high hemolymph flow and hemocyte concentration. (A–F) Dissected larval (A–C) and adult (D–F) dorsal abdomens with fluorescently labeled hemocytes (CM-DiI; red) at 4 h after injection with fluorescent microspheres (green). In larvae, microspheres preferentially aggregated in the eighth abdominal segment (A), where there is a high concentration of hemocytes (B, circle in C). In adults, microspheres preferentially aggregated at the periostial regions of the heart (D), where the periostial hemocytes are located (E, arrows in F). (G–L) Dissected larval (G–I) and adult (J–L) dorsal abdomens with fluorescently labeled hemocytes at 24 h after injection with fluorescent microspheres, showing an aggregation pattern identical to that observed at 4 h post-treatment. Directional arrows: A anterior, P posterior, L lateral. (PDF 1117 kb) [file 12915_2016_305_MOESM5_ESM.pdf]

Functional integration of the circulatory, immune, and respiratory systems in mosquito larvae: pathogen killing in the hemocyte-rich tracheal tufts

Garrett P. League and Julián F. Hillyer (julian.hillyer@vanderbilt.edu)

Department of Biological Sciences, Vanderbilt University, Nashville, TN, U.S.A.

BMC Biology, 2016

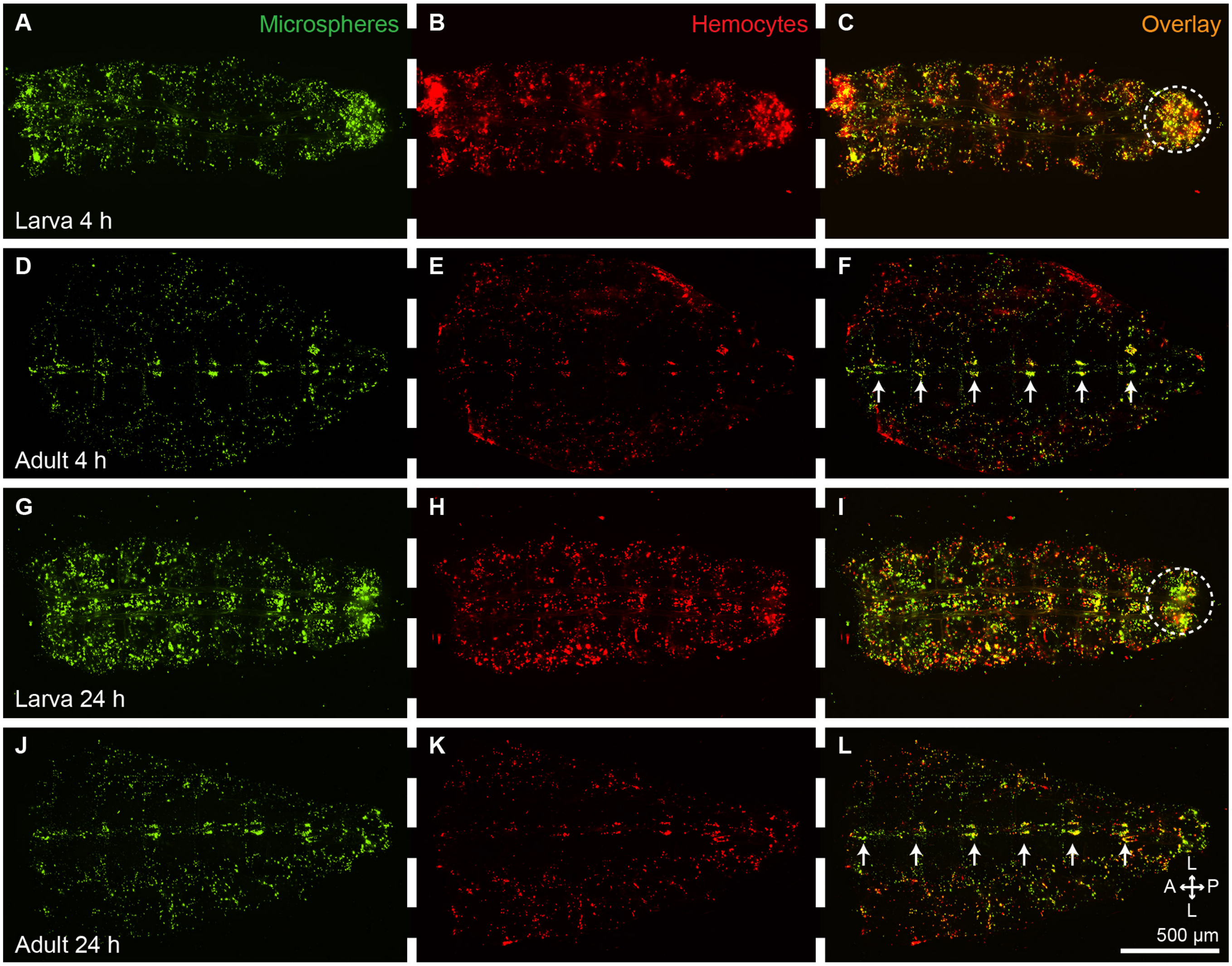

Additional File 5: Figure S3. Microspheres injected into larvae and adults aggregate in regions of high hemolymph flow and hemocyte concentration. (**A-F**) Dissected larval (A-C) and adult (D-F) dorsal abdomens with fluorescently labeled hemocytes (CM-DiI; red) at 4 h after injection with fluorescent microspheres (green). In larvae, microspheres preferentially aggregated in the 8th abdominal segment (A), where there is a high concentration of hemocytes (B, circle in C). In adults, microspheres preferentially aggregated at the periostial regions of the heart (D), where the periostial hemocytes are located (E, arrows in F). (**G-L**) Dissected larval (G-I) and adult (J-L) dorsal abdomens with fluorescently labeled hemocytes

at 24 h after injection with fluorescent microspheres, showing an aggregation pattern identical to that observed at 4 h post-treatment. Directional arrows: A, anterior; P, posterior; L, lateral.
