# Supplementary material for: Functional integration of the circulatory, immune, and respiratory systems in mosquito larvae: pathogen killing in the hemocyte-rich tracheal tufts
Source: BMC Biol. 2016 Sep 19;14:78. doi: 10.1186/s12915-016-0305-y (PMC5027632; doi:10.1186/s12915-016-0305-y)
Supplement: Additional file 6: Figure S4. — E. coli injected into larvae and adults aggregate in regions of high hemolymph flow and hemocyte concentration. (A–F) Dissected larval (A–C) and adult (D–F) dorsal abdomens with fluorescently labeled hemocytes (CM-DiI; red) at 4 h after injection with GFP-E. coli (green). In larvae, E. coli preferentially aggregated in the eighth abdominal segment (A), where there is a high concentration of hemocytes (B, circles in C). In adults, E. coli aggregated at the periostial regions of the heart (D), where the periostial hemocytes are located (E, arrows in F). (G–L) Dissected larval (G–I) and adult (J–L) dorsal abdomens with fluorescently labeled hemocytes at 24 h after injection with GFP-E. coli. The aggregation pattern of E. coli in adults at 24 h after treatment was similar to that observed at 4 h post-treatment, but in larvae, fluorescence from E. coli was not observed anywhere in the body because the infection had been largely cleared. Diagonal lines in panels G–I denote the edges of rotated images. Directional arrows: A anterior, P posterior, L lateral. (PDF 679 kb) [file 12915_2016_305_MOESM6_ESM.pdf]

Functional integration of the circulatory, immune, and respiratory systems in mosquito larvae: pathogen killing in the hemocyte-rich tracheal tufts

Garrett P. League and Julián F. Hillyer (julian.hillyer@vanderbilt.edu)

Department of Biological Sciences, Vanderbilt University, Nashville, TN, U.S.A.

BMC Biology, 2016

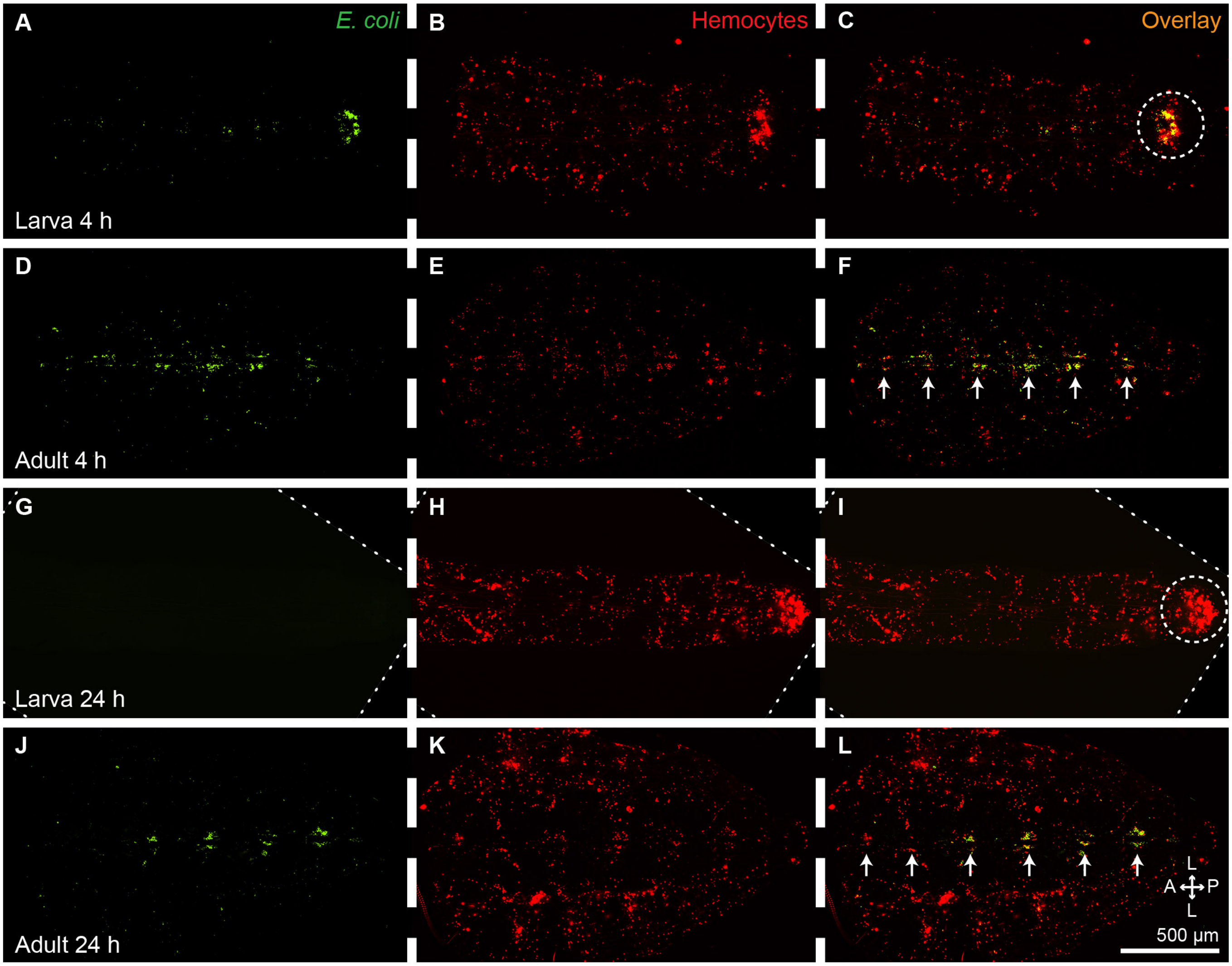

Additional File 6: Figure S4. ***E. coli*** **injected into larvae and adults aggregate in regions of high hemolymph flow and hemocyte concentration.** (**A-F**) Dissected larval (A-C) and adult (D-F) dorsal abdomens with fluorescently labeled hemocytes (CM-DiI; red) at 4 h after injection with GFP-*E. coli* (green). In larvae, *E. coli* preferentially aggregated in the 8th abdominal segment (A), where there is a high concentration of hemocytes (B, circles in C). In adults, *E. coli* aggregated at the periostial regions of the heart (D), where the periostial hemocytes are located (E, arrows in F). (**G-L**) Dissected larval (G-I) and adult (J-L) dorsal abdomens with fluorescently labeled hemocytes at 24 h after injection with GFP-*E. coli*. The aggregation pattern of *E. coli* in adults at 24 h after treatment was similar to that observed at 4 h post-

treatment, but in larvae, fluorescence from *E. coli* was not observed anywhere in the body because the infection had been largely cleared. Diagonal lines in panels G-I denote the edges of rotated images. Directional arrows: A, anterior; P, posterior; L, lateral.
