# Supplementary material for: Functional integration of the circulatory, immune, and respiratory systems in mosquito larvae: pathogen killing in the hemocyte-rich tracheal tufts
Source: BMC Biol. 2016 Sep 19;14:78. doi: 10.1186/s12915-016-0305-y (PMC5027632; doi:10.1186/s12915-016-0305-y)
Supplement: Additional file 7: Figure S5. — Fluorescence intensity and optical density at the larval periostial regions and tracheal tufts were measured using custom-drawn regions of interest. (A–C) Representative fluorescence and bright-field images of dissected dorsal abdomens from larvae infected with GFP-E. coli for 4 h (A) and 24 h (B), and from a naïve larva (C). Images such as these were used to quantify mean fluorescence intensity of GFP-E. coli (A) and CM-DiI-stained hemocytes (C), as well as the mean optical density (OD) of melanin deposits (B). Custom regions of interest (ROIs) 1–7 contain each periostial region (PR), delineated as the area in each abdominal segment that lies between the dorsal longitudinal tracheal trunks and stretches from the abdominal suture to the dorsal abdominal tracheal commissure. ROIs 8 and 9 contain the left and right tracheal tufts (TT). Directional arrows: A anterior, P posterior, L lateral. (PDF 449 kb) [file 12915_2016_305_MOESM7_ESM.pdf]

Functional integration of the circulatory, immune, and respiratory systems in mosquito larvae: pathogen killing in the hemocyte-rich tracheal tufts

Garrett P. League and Julián F. Hillyer (julian.hillyer@vanderbilt.edu)

Department of Biological Sciences, Vanderbilt University, Nashville, TN, U.S.A.

BMC Biology, 2016

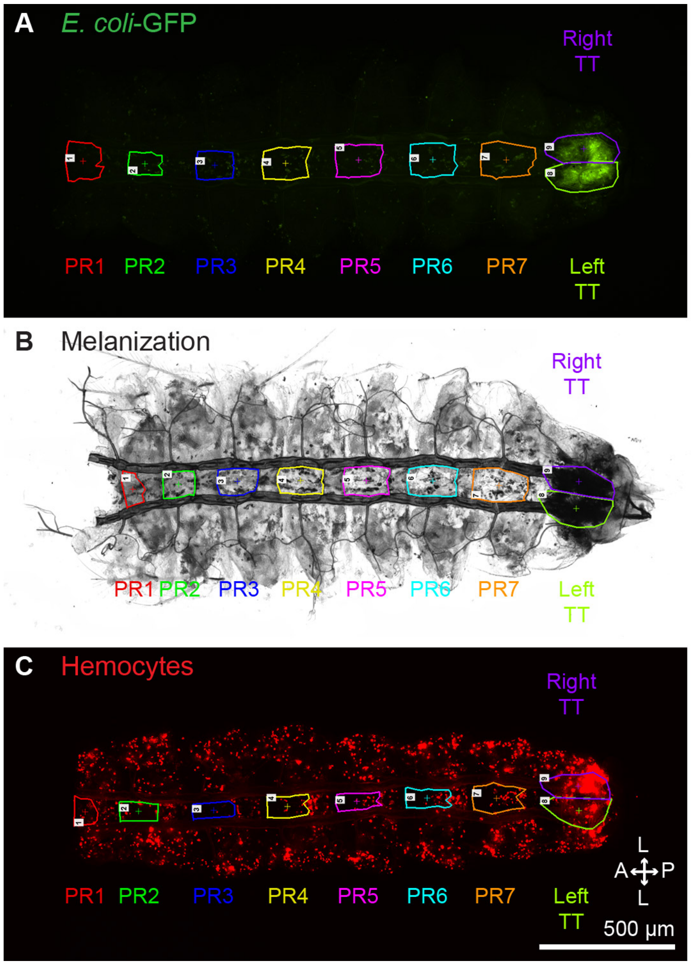

Additional File 7: Figure S5. Fluorescence intensity and optical density at the larval periostial regions and tracheal tufts were measured using custom drawn regions of interest. (**A-C**) Representative fluorescence and bright-field images of dissected dorsal abdomens from larvae infected with GFP-*E. coli* for 4 h (A) and 24 h (B), and from a naïve larva (C). Images such as these were used to quantify mean fluorescence intensity of GFP-*E. coli* (A) and CM-DiI-stained hemocytes (C), as well as the mean optical density (OD) of melanin deposits (B). Custom regions of interest (ROIs) 1-7 contain each periostial region (PR), delineated as the area in each abdominal segment that lies between the dorsal longitudinal tracheal trunks and stretches from the abdominal suture to the dorsal abdominal tracheal commissure. ROIs 8-9 contain the left and right tracheal tufts (TT). Directional arrows: A, anterior; P, posterior; L, lateral.
