# Supplementary material for: Functional integration of the circulatory, immune, and respiratory systems in mosquito larvae: pathogen killing in the hemocyte-rich tracheal tufts
Source: BMC Biol. 2016 Sep 19;14:78. doi: 10.1186/s12915-016-0305-y (PMC5027632; doi:10.1186/s12915-016-0305-y)
Supplement: Additional file 8: Figure S6. — GFP signal from periostial region ROIs of larvae originate from pericardial cells and segmental hemocytes. (A–C) Fluorescence images taken at 4 h post-treatment showing that pericardial cells (A; IgG 568 nm, red) pinocytose GFP (B) that is flowing with the hemolymph following its release from degrading GFP-E. coli (overlay of A and B is shown in C). (D–F) Bright-field and fluorescence images of a portion of the seventh and eighth abdominal segments of a dissected larva at 4 h post-injection with GFP-E. coli (green), showing that both the tracheal tuft hemocytes and the dorsal segmental hemocytes (CM-DiI; red) engage in phagocytosis. A group of segmental hemocytes (D, E, rectangle) that have phagocytosed E. coli within the seventh abdominal segment region of interest (area within dashed outline) is magnified in F. Neither pathogens nor hemocytes were detected within the periostial regions. Directional arrows: A anterior, P posterior, L lateral. (PDF 471 kb) [file 12915_2016_305_MOESM8_ESM.pdf]

Functional integration of the circulatory, immune, and respiratory systems in mosquito larvae: pathogen killing in the hemocyte-rich tracheal tufts

Garrett P. League and Julián F. Hillyer (julian.hillyer@vanderbilt.edu)

Department of Biological Sciences, Vanderbilt University, Nashville, TN, U.S.A.

BMC Biology, 2016

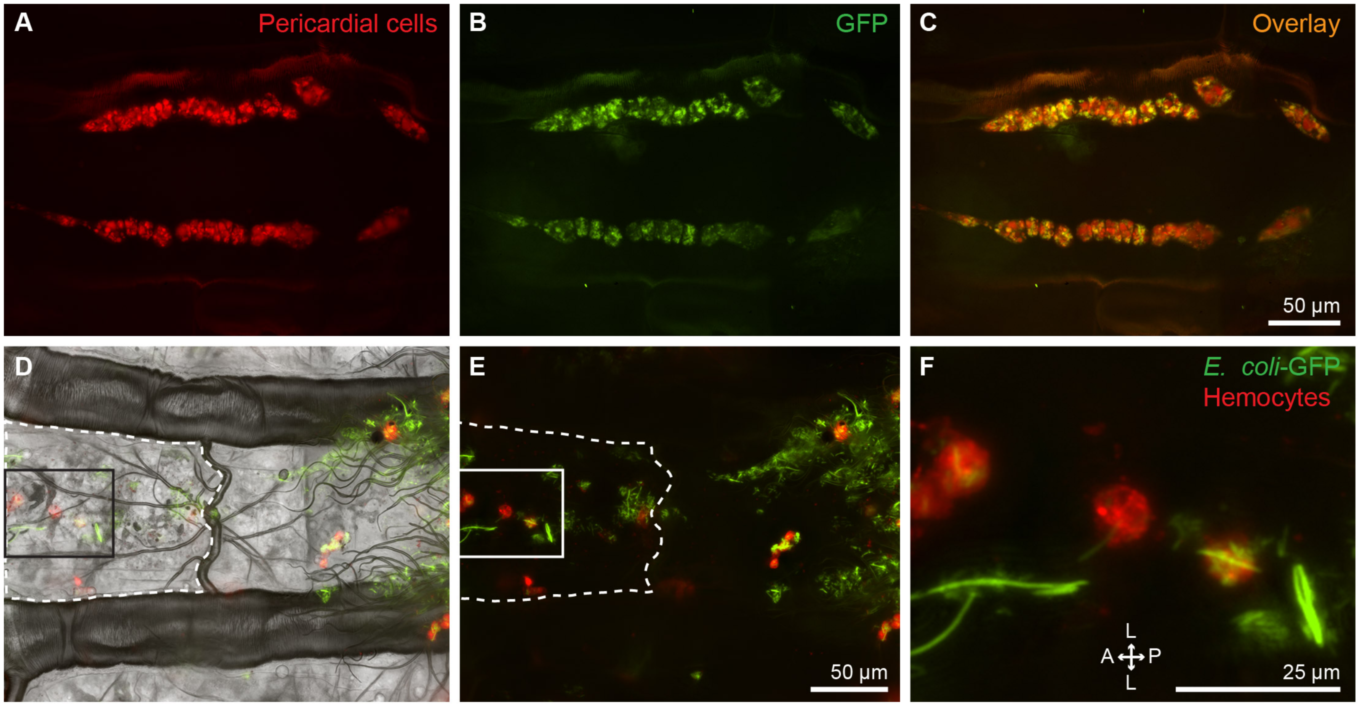

Additional File 8: Figure S6. GFP signal from periostial region ROIs of larvae originate from pericardial cells and segmental hemocytes. (**A-C**) Fluorescence images taken at 4 h post-treatment showing that pericardial cells (A; IgG 568 nm, red) pinocytose GFP (B) that is flowing with the hemolymph following its release from degrading GFP-*E. coli* (overlay of A and B is shown in C). (**D-F**) Bright-field and fluorescence images of a portion of the 7th and 8th abdominal segments of a dissected larva at 4 h post-injection with GFP-*E. coli* (green), showing that both the tracheal tuft hemocytes and the dorsal segmental hemocytes (CM-DiI; red) engage in phagocytosis. A group of segmental hemocytes (D-E, rectangle) that have phagocytosed *E. coli* within the 7th abdominal segment region of interest (area within dashed outline) is magnified in F. Neither pathogens nor hemocytes were detected within the periostial regions. Directional arrows: A, anterior; P, posterior; L, lateral.
