# Supplementary material for: Functional integration of the circulatory, immune, and respiratory systems in mosquito larvae: pathogen killing in the hemocyte-rich tracheal tufts
Source: BMC Biol. 2016 Sep 19;14:78. doi: 10.1186/s12915-016-0305-y (PMC5027632; doi:10.1186/s12915-016-0305-y)
Supplement: Additional file 9: Figure S7. — Tracheal tuft hemocytes phagocytose melanized bacteria. (A, B) Fluorescence (A) and bright-field overlay (B) images of tracheal tuft hemocytes (CM-DiI; red) phagocytosing aggregates of melanized (black) GFP-E. coli (green) at 4 h post-infection. Notice that melanin deposits dampen the fluorescence signal. Directional arrows: A anterior, P posterior, L lateral. (PDF 232 kb) [file 12915_2016_305_MOESM9_ESM.pdf]

Functional integration of the circulatory, immune, and respiratory systems in mosquito larvae: pathogen killing in the hemocyte-rich tracheal tufts

Garrett P. League and Julián F. Hillyer (julian.hillyer@vanderbilt.edu)

Department of Biological Sciences, Vanderbilt University, Nashville, TN, U.S.A.

BMC Biology, 2016

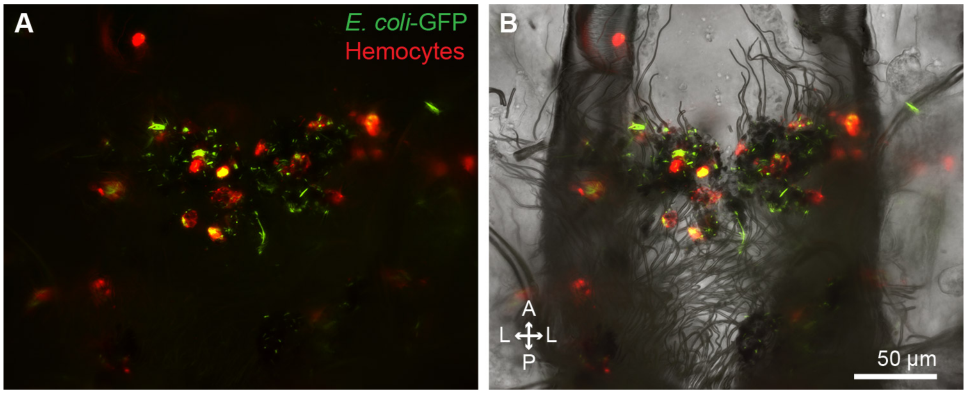

Additional File 9: Figure S7. Tracheal tuft hemocytes phagocytose melanized bacteria. (**A-B**) Fluorescence (A) and bright-field overlay (B) images of tracheal tuft hemocytes (CM-DiI; red) phagocytosing aggregates of melanized (black) GFP-*E. coli* (green) at 4 h post-infection. Notice that melanin deposits dampen the fluorescence signal. Directional arrows: A, anterior; P, posterior; L, lateral.
